# Contaminants of emerging concern in tributaries to the Laurentian Great Lakes: II. Biological consequences of exposure

**DOI:** 10.1371/journal.pone.0184725

**Published:** 2017-09-27

**Authors:** Linnea M. Thomas, Zachary G. Jorgenson, Mark E. Brigham, Steven J. Choy, Jeremy N. Moore, Jo A. Banda, Daniel J. Gefell, Thomas A. Minarik, Heiko L. Schoenfuss

**Affiliations:** 1 Aquatic Toxicology Laboratory, St. Cloud State University, St. Cloud, Minnesota, United States of America; 2 U.S. Fish & Wildlife Service, Bloomington, Minnesota, United States of America; 3 U.S. Geological Survey, Mounds View, Minnesota, United States of America; 4 U.S. Fish and Wildlife Service, Madison, Wisconsin, United States of America; 5 U.S. Fish and Wildlife Service, East Lansing, Michigan, United States of America; 6 U.S. Fish and Wildlife Service, Columbus, Ohio, United States of America; 7 U.S. Fish and Wildlife Service, Cortland, New York, United States of America; 8 Metropolitan Water Reclamation District of Greater Chicago, Cicero, Illinois, United States of America; Northwest Fisheries Science Center, UNITED STATES

## Abstract

The Laurentian Great Lakes contain one fifth of the world’s surface freshwater and have been impacted by human activity since the Industrial Revolution. In addition to legacy contaminants, nitrification and invasive species, this aquatic ecosystem is also the recipient of Contaminants of Emerging Concern (CECs) with poorly understood biological consequences. In the current study, we documented the presence, concentrations, and biological effects of CECs across 27 field sites in six Great Lakes tributaries by examining over 2250 resident and caged sunfish (*Lepomis ssp*.) for a variety of morphological and physiological endpoints and related these results to CEC occurrence. CEC were ubiquitous across studies sites and their presence and concentrations in water and sediment were highest in effluent dominated rivers and downstream of municipal wastewater treatment plant discharges. However, even putative upstream reference sites were not free of CEC presence and fish at these sites exhibited biological effects consistent with CEC exposure. Only the Fox River exhibited consistent adverse biological effects, including increased relative liver size, greater prominence of hepatocyte vacuoles and increased plasma glucose concentrations. Canonical Redundancy Analysis revealed consistent patterns of biological consequences of CEC exposure across all six tributaries. Increasing plasma glucose concentrations, likely as a result of pollutant-induced metabolic stress, were associated with increased relative liver size and greater prominence of hepatocyte vacuoles. These indicators of pollutant exposure were inversely correlated with indicators of reproductive potential including smaller gonad size and less mature gametes. The current study highlights the need for greater integration of chemical and biological studies and suggests that CECs in the Laurentian Great Lakes Basin may adversely affect the reproductive potential of exposed fish populations.

## Introduction

The Laurentian Great Lakes contain one fifth of the world’s surface freshwater and are home to diverse aquatic and terrestrial habitats that support a large number of fish and wildlife resources. Throughout the Great Lakes, multiple areas have been designated important for resource management, including National Wildlife Refuges (https://www.fws.gov/refuges) and National Estuarine Research Reserves (https://coast.noaa.gov/nerrs). The Great Lakes are also an important economical resource due to recreational and commercial fishing as well as multiple commercial ports. These ports were instrumental in the expansion of manufacturing during the Industrial Revolution in North America. The detrimental environmental legacy of the Industrial Revolution in urban centers around the Great Lakes has been the focus of toxicological studies for many years [1], including recently, studies of Contaminants of Emerging Concern (CECs) [2] which may interact with molecular pathways in exposed organisms [3–6]. Numerous laboratory and a limited number of field studies have identified consequences of CEC exposures ranging from molecular to organismal and population-level effects [7–21]. These compounds, including pharmaceuticals, personal care products, pesticides and industrial chemicals enter aquatic environments through agricultural and urban storm water runoff, on-site septic system discharge, and outflows from municipal wastewater treatment plants and industrial sources [7, 22–36].

The multitude of CEC sources result in complex mixtures [37] that challenge our ability to predict effects to biota based on chemistry alone. Consequently, the evaluation of biological effects of CEC exposure in fish populations requires an integrated approach to acknowledge the complexity of the associated aqueous chemistry and fish biology. The objectives of this study were two-fold. First, we examined the presence and severity of biological effects commonly associated with exposure to CECs. Second, we assessed whether biological effects in resident and caged sunfish correlated with the presence of CECs in the adjacent aquatic environment.

**Fig 1:**
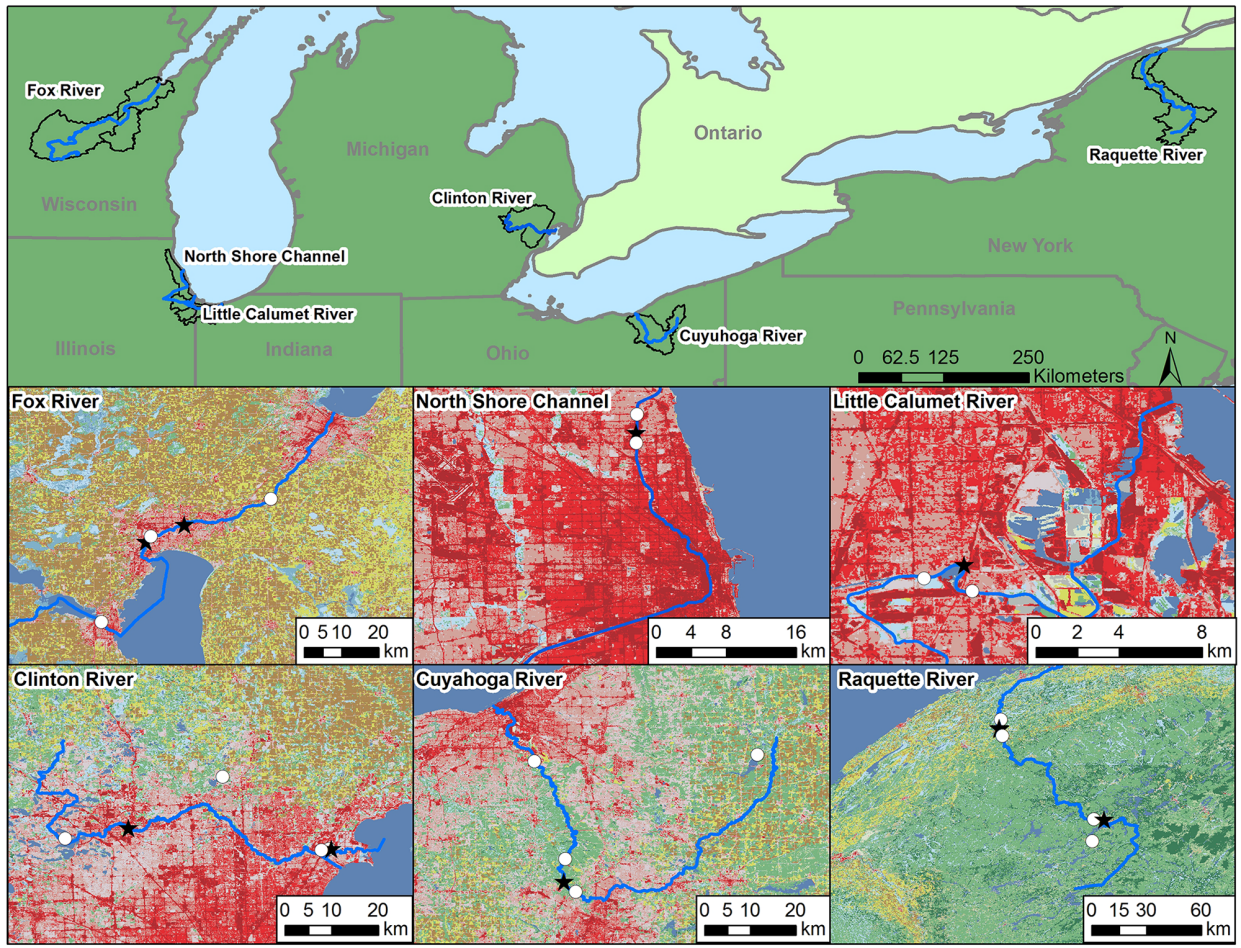
Location of sample sites from 2013 and 2014. For detailed site information, see Table 1. River basins are outlined on overview map (top). Maps of the six river watersheds are highlighted below. White circles indicate approximate resident fish collection and caging sites in each river. Stars indicate approximate resident fish collection and caging sites in each river that are immediately below wastewater discharge outfalls. Land use is indicated with light- and medium-grey patches of forest and agricultural and dark gray urban areas with linear road features. Map was prepared using ArcGIS 10.3.1 software by Esri.

We selected six Great Lakes tributaries for the current study to include the array of land use patterns common in the Great Lakes Basin (Fig 1). These land uses ranged from predominately rural, including forest interspersed with agriculture and limited municipalities, to intensely agricultural with limited urban influence, to exclusively urban. Most rivers contained a range of influences across their watersheds and, whenever land use allowed, a putative reference site (usually in the headwaters) was included. Concurrent resident and caged sunfish (*Lepomis* ssp.) assessments were used to more fully assess potential effects of CEC exposures to fish [38] (Fig 1).

The resultant multi-dimensional matrix of chemical, biological, and geographic data required an integrative multivariate model to identify relationships between two sets of variables, chemical occurrence and biological consequences. Canonical analyses, a permutation of principal component analyses, provide frequently used tools in community ecology to reduce the multidimensionality of data matrices and identify differences and relationships between groups of samples [39]. For this study, Canonical Redundancy Analysis (RDA) was used with CEC concentration as explanatory variables and biological effects as response variables [40]. Using RDA, the relationship between both sets of variables can be evaluated by determining how much variation in the response variables can be explained by the set of explanatory variables. This is accomplished by regressing multiple response variables onto multiple explanatory variables [41]. The resultant matrix is then analyzed using a principal component analysis. However, while relationships may be observed between the groups, this analysis cannot attribute causality.

We expected that CEC concentrations would be higher downstream of wastewater treatment plant outfalls and lower at upstream sites, with resident and caged fish showing increasing evidence of CEC exposure along a downstream gradient. Concurrently, we expected that an increase in the hepatosomatic index, liver hepatocyte vacuolization, glucose concentrations, and pathologies would be associated with an increase in CEC concentrations. Condition factor, gonadosomatic index and gonadal maturity would decline as CEC concentrations increase. Plasma vtg concentrations are likely to increase with increasing CEC concentrations, however, since many CECs are not estrogenic, this prediction may not accurately reflect the complexity of CEC mixture effects. When combined, these two objectives allow for the largest study to-date examining the biological consequences of CEC exposure on resident and caged fish in the Laurentian Great Lakes tributaries.

## Materials and methods

### Ethics statement

Permission for access to field sites and scientific collectors permits were secured by the U.S. Fish and Wildlife Service. All resident fish collections occurred on public land and did not involve any protected species. This study was carried out in strict accordance with the recommendations in the Guide for the Care and Use of Laboratory Animals of the National Institutes of Health. The protocol was approved by the Institutional Animal Care and Use Committees of St. Cloud State University (permit number: 0213). Fish were maintained in aerated coolers filled with river water until they could be processed (usually within eight hours). Fish were euthanized by a sharp blow to the head or by immersion in the fish anesthetic MS-222. All efforts were made to minimize distress to the animals.

### Study area and design

A total of six rivers, containing 27 study sites, were sampled over two years (Fig 1; Table 1) and represented an array of land uses common in the Great Lakes watershed. For the purposes of the current study, three stream reaches (upstream, middle, downstream) were identified for the collection of resident sunfish (six rivers x three reaches = 18 stream reaches). Furthermore, within each stream reach, we identified at least one and as many as three study sites (for a total of 27 study sites) for the caging of hatchery-reared sunfish and the collection of water and sediment samples (Table 1). Land uses were evaluated using the National Land Cover Database [42]. Land use categories were combined into the more general groups of Urban, Forest, Grass, Crop, Water, and Other [37]. For every river basin, the total number of pixels for each land use category were determined to calculate the area of each land use, the areas of each land use category were summed, and the percentage of each land use category was determined within each basin.

**Table 1:** Sample site information.

River (year)	Basin (km^2^)	Land use (percent)					Reach section	Site	Latitude	Longitude	Km to next downstream site (km to river mouth)	Dams to next upstream site (#)	Point sources to next upstream site (#)
		Urban	Forest	Grass	Crop	Water							
**Fox River, WI (2014)**	7,385	9	13	4	50	10	Upstream	LBM-1	44.035444	-88.565694	39 (96)	0	0
							Middle	LLB-3	44.229694	-88.458750	7.1 (57)	0	1
								LLB-5	44.242222	-88.446361	2.8 (49.9)	1	0
							Downstream	LLB-6	44.269944	-88.365694	22.5 (47.05)	1	1
								FXR-13	44.333056	-88.156389	24.6 (24.6)	0	0
**N. Shore Channel, Chicago River, IL (2014)**	695	84	4	10	1	1	Upstream	CHI-112	42.040861	-87.709778	2.1 (22.4)	N/A	0
							Middle	CHI-RP4	42.021806	-87.710417	1.3 (20.3)	0	1
							Downstream	CHI-36	42.011778	-87.710389	19 (19)	0	0
**Little Calumet River, IL (2014)**	1,470	54	8	10	16	1	Upstream	CHI-56	41.650917	-87.615583	17.7 (17.7)	N/A	0
							Middle	CHI-RP7	41.662361	-87.619083	1.8 (19.5)	0	1
							Downstream	CHI-76	41.656528	-87.636472	1.6 (21.1)	0	1
**Clinton River, MI (2013)**	2,065	39	14	15	19	3	Upstream	CLI-5	42.617306	-83.373944	16.4 (90.5)	N/A	0
							Middle	CLI-4	42.736083	-83.071889	52.6 (63.7)	N/A	0
							Downstream	CLI-3	42.637444	-83.252944	63 (74.1)	1	1
								CLI-2	42.597694	-82.867250	0.4 (11.1)	2	1
								CLI-1	42.597056	-82.862583	10.7 (10.7)	0	1
**Cuyahoga River, OH (2013/14)**	2,100	21	30	18	15	2	Upstream	CUY-5	41.387222	-81.200472	66.2 (133.4)	N/A	0
							Middle	CUY-4	41.135194	-81.546972	4.9 (67.2)	8	40
								CUY-3	41.159944	-81.572833	6.9 (62.3)	0	1
								CUY-2	41.200861	-81.568556	33.7 (55.4)	0	0
							Downstream	CUY-1	41.393944	-81.629778	21.7 (21.7)	0	0
**Raquette River, NY (2013/14)**	3,260	2	69	1	3	8	Upstream	RAQ-6	44.125162	-74.538418	13.6 (169.4)	N/A	0
							Middle	RAQ-5	44.231799	-74.479225	4.3 (155.8)	0	1
								RAQ-4	44.233938	-74.529803	94.5 (151.5)	1	0
							Downstream	RAQ-3	44.679900	-74.994173	0.4 (57)	13	0
								RAQ-2	44.682754	-74.996702	6.1 (56.6)	0	2
								RAQ-1	44.723843	-74.989601	50.5 (50.5)	1	0

Sample year, basin drainage area (km^2^), estimated land use in the drainage basin (as percent of total land use), river reach section, sample site identifier, latitude and longitude, distance (in km) from each site to the next downstream site (including total distance to river mouth at a Great Lake), number of dams between that site and the next site upstream, and the number of identified point sources (e.g., wastewater treatment plants or combined sewer overflows) between that site and the next site upstream. Due to the complex and managed hydrology of the Chicago Area waterways system, distance from Lake Michigan for Little Calumet River sites do not imply an intuitive upstream—downstream flow direction.

Limitations to migration, including dams or sufficient distance between reaches, were used to identify reaches of likely distinct *Lepomis* populations. For all rivers, the middle reach was a recipient of treated wastewater from a wastewater treatment plant (however, other river reaches could also be recipients of treated wastewater, see Table 1). Fish were caged at three to six sites in each river across the three reaches to represent each reach as well as specific potential point sources. Where logistically feasible we placed a cage in the discharge zone of major wastewater treatment plants, as well as upstream and downstream of these plants (Table 1, Fig 1). Whenever land use permitted (i.e., upstream reaches with little shoreline development), an upstream putative reference site was included.

The Cuyahoga River (Ohio) and Raquette River (New York) were sampled in 2013 and 2014 to examine annual changes in CEC presence and biological effects. The Clinton River (Michigan) was sampled in 2013, while the Lower Wolf and Upper Fox Rivers (Wisconsin—hereafter “Fox River”), the North Shore Channel of the Chicago River (Illinois–hereafter “Chicago River”), and the Little Calumet River (Illinois) were sampled in 2014 (S1 Table).

The Fox River is dominated by cropland and dairy farming with small towns located along the river. The intensity of agricultural land use in this watershed precluded the identification of a suitable reference site. The north branch of the Chicago River is densely urban with study sites upstream, at, and immediately downstream of a major municipal wastewater treatment plant. The Little Calumet River is a commercial shipping waterway located just south of Chicago, IL. Land use in the basin is densely urban and industrial with minor cropland, forest or grassland. The middle reach is located immediately downstream of a major municipal wastewater treatment plant, while the other two sites are upstream and downstream of the plant. The Chicago and Little Calumet Rivers both consist primarily of wastewater effluent from the multiple discharge stations located along the rivers. The Clinton River basin, located northeast of Detroit, contains a mix of forest, grass, and cropland in the northern section of the drainage basin and primarily urban land use farther south. The upper reach is above identified point sources but is surrounded by urban land uses. The middle and downstream are influenced by multiple municipal treatment plants. No suitable reference sites could be located in the intensively used Chicago, Little Calumet, and Clinton Rivers. The lack of suitable reference sites had been noted previously [4] for the former two rivers. The Cuyahoga River represents a mix of urban, agricultural, and forested land uses. We identified a primarily forested upstream reach as a putative reference site. Its middle reach is influenced by combined sewer overflows located just downstream of the city of Akron and a major municipal treatment plant. The Raquette River is forested with occasional dairy farming and some municipalities along the river. The upstream reach is expected to be minimally impacted and was identified as a putative reference site, while the middle reach is located just downstream of the Tupper Lake municipal wastewater treatment facility. The downstream reach contained sites that bracketed the Potsdam, NY municipal wastewater treatment plant.

### Water sample collection

The U.S. Geological Survey (USGS) collected surface water samples at each site twice during each sampling period, near the beginning and end of the caged fish exposures. Sediment samples were collected once from each site. Detailed sampling and analytical methods, along with a summary of quality-control data, are published elsewhere [43, 44]. Briefly, water samples were collected in 1-L, baked amber glass bottles using a depth-integrated sampling method with a weighted-bottle sampler. The most recently-deposited bottom sediments (top 10 cm) were sampled using a stainless-steel Ekman dredge. All equipment was stainless steel or other inert material. Water and sediment samples were shipped on ice to the USGS National Water Quality Laboratory.

### Chemical assessment

At the USGS National Water Quality Laboratory, water samples were analyzed for over 200 chemicals. For wastewater indicators (e.g., fragrances, flame retardants, plasticizers, and domestic pesticides), unfiltered water samples were analyzed by continuous liquid-liquid extraction and capillary-column gas chromatography/mass spectrometry (GC/MS) following published methods [45]. Steroid hormones, sterols, and bisphenol A were analyzed in unfiltered water samples by solid-phase extraction, derivatization, and gas chromatography with tandem mass spectrometry (GC/TMS) following the methods in Foreman et al. [46]. Filtered water samples were analyzed for pharmaceuticals by direct aqueous injection high-performance liquid chromatography with tandem mass spectrometry following published methods [47].

Sediment samples were analyzed for over 100 chemicals. For wastewater indicators, sediment samples were dried and solvent-extracted. Extracts were cleaned with a solid-phase extraction, and then chemical concentrations sediment samples were dried and solvent-extracted. Extracts were cleaned with a solid-phase extraction, and then chemicals were determined by capillary-column GC/MS following methods in Burkhardt et al. [48]. For steroid hormones, sterols, and bisphenol A, sediment samples were analyzed by adapting the methods in Burkhardt et al. [48], which are described in Fischer et al. [49]. Accelerated solvent extraction techniques were used to extract pharmaceuticals and antidepressants from sediment samples. High performance liquid chromatography, as described in Lee et al. [50], was then used to determine concentrations of pharmaceuticals and antidepressants. All chemical data, including quality assurance and quality control information are previously published [43,44].

### Biological sample collection

Concurrent resident and caged fish assessments were used to evaluate differences in effects two ways. The first was duration of exposure through the use of short term exposed caged fish and long term exposed resident fish. The second was differences in continuous exposure (caged fish confined to the exposure area) to periodic exposure (in-stream movement of resident fish). Resident sunfish (genus *Lepomis*) were collected during the spawning season in spring 2013 and/or spring 2014 using standard boat electro-shocking equipment and transferred to processing areas using aerated holding tanks. An attempt was made to collect 40 sunfish in the genus *Lepomis* at each river reach [38] as they are native and locally abundant throughout the Great Lakes watershed and maintain site fidelity during the spring when males guard nest sites [51, 52]. While sunfish are common, the abundance of each species often differs widely between reaches and collection events. As a consequence, we combined the most commonly found sunfish species, bluegill (*Lepomis macrochirus*), pumpkinseed sunfish (*Lepomis gibbosus*), green sunfish (*Lepomis cyanellus*) and redear sunfish *(Lepomis microlophus*) for the analysis of the effects of CECs on resident sunfish [4]. This approach is justified by the shared habitat of these species, their phylogenetic closeness [53, 54] their frequent hybridization [53, 55] and the fertility of hybrid offspring [56].

Sunfish were sacrificed on site or at nearby laboratory facilities within eight hours of their collection. Whole body wet weights (0.01g precision) and length were measured for each fish. Blood was drawn from the caudal vasculature and centrifuged at 5,000 g for 8 min to separate plasma. Gonads and livers from each fish were excised and immediately weighed as wet weight (0.001 g precision, Mettler Toledo AG245, Columbus, OH). Care was taken to weigh organs quickly to avoid desiccation while keeping the organs away from any fluid that would further wet the tissue. Three to five tissue samples, each approximately 3 mm^3^ in volume, were collected from each organ and placed into histology specimen cassettes, immersed in 10% neutral buffered formalin, and shipped to St. Cloud State University for histological processing. Plasma samples were shipped on dry ice to St. Cloud State University and stored at -80°C until analysis.

Similar to other studies using caged fish as sentinels for environmental exposures in aquatic environments [26, 57, 58, 59], at least 40 hatchery-reared sunfish were caged and deployed at multiple sites per river (Table 1, Fig 1) for 14 days concurrent with resident fish collections. Prior to deployment, a subsample of sunfish was processed to determine baseline conditions and to assure that fish were mature at the time of deployment. Fish were transported in aerated coolers from nearby hatcheries to the field sites, placed in cylindrical cages (1 m length x 0.4 m diameter) made of plastic mesh (1cm mesh size) reinforced with PVC pipe and anchored to the bottom of the waterway, ensuring that the fish cage was in direct contact with the bottom sediment [4]. Two replicate cages were deployed at each site, each containing 25 mixed-sex bluegill sunfish (hybrid bluegill in the Raquette River in 2013 due to a lack of bluegill sunfish). After retrieving the cages, fish were placed in aerated coolers and processed as described for resident sunfish (see above).

### Biological assessment

#### Morphometric indices

Body weight and total length were used to calculate the condition factor (CF = body weight/[total length]^3^), a measure of the overall health of the fish [60]. Gonad and whole-body weights were used to calculate the gonadosomatic index (GSI = gonad weight/whole body weight X 100). Liver and whole-body weights were used to calculate the hepatosomatic index (HSI = liver weight/whole body weight X 100).

#### Histological analysis

For histological assessments, tissue samples were fixed for at least 1 week, dehydrated through a series of ethanol and xylene baths in a Leica automated tissue processor TP 1050 (Leica, Wetzlar, Germany) and embedded in paraffin using a Thermo Scientific Microm EC 350–1 embedding station (Waltham, MA). A previous study [61] demonstrated that a central location and greater number of tissue sections taken from the testes of smallmouth bass was positively correlated with the probability to detect intersex in this organ. Consequently, embedded tissues were sectioned at approximately 1/3 and 2/3 of the depth of the sample (resulting in tissue slices ~ 100 μm apart) using a Reichert-Jung cassette microtome (Leica, Wetzlar, Germany; 5 μm sections).

At least six sections from each organ (gonad, liver) were stained using standard hematoxylin and eosin techniques [62, 63] in a Leica Autostainer XL, similar to methods used in other studies [12, 64, 65]. Histological sections were assessed by an experienced histologist (HLS) and ranked on a semi-quantitative scale (1–4) for vacuolization of liver hepatocytes (1—vacuoles visible in <5% of total area; 2—vacuoles small but throughout image in <25% of area; 3—broad presence of large vacuoles in 25%-50% of area; 4 –vacuoles prominent covering more than 50% of the field of view) (S1 Fig). The developmental stage of the gonad (testis or ovary) was also evaluated based on the proportion of cell types visible in the field of view (female: perinuclear oocyte, cortical alveolar, early vitellogenic, late vitellogenic; male: spermatogonia, spermatocyte, spermatid, and spermatozoa) (S2 Fig). The overall maturity of the sample (on a scale of 1 = immature to 4 = only mature sperm present) was calculated as:

$$\text{Testis}=\left(\left({\%}_{\text{spermatogonia}}\right)+\left({\%}_{\text{spermatocytes}}\text{ x }2\right)+\left({\%}_{\text{spermatids}}\text{ x }3\right)+\left({\%}_{\text{spermatozoa}}\text{x }4\right)\right)/100$$

$$\text{Ovary}=\left(\left({\%}_{\text{primary oocyte}}\right)+\left({\%}_{\text{cortical alveolar}}\text{ x }2\right)+\left({\%}_{\text{vitellogenic}}\text{ x }3\right)+\left({\%}_{\text{mature}}\text{ x }4\right)\right)/100$$

Seven macroscopic and microscopic pathological observations (1 = pathology present; 0 = pathology absent for each pathological category) were summed for each fish to generate a pathology score (0 = no observed pathologies; 7 = all pathology categories represented in sampled fish). All macroscopic pathologies (lesions, deformities, missing structures, parasites visible without magnification) in livers and gonads were combined into one value as these observations were uncommon. For liver and gonad separately, we recorded the microscopic presence of eosinophilic fluids, parasitic cysts and/or other pathologies (to include intersex, atretic oocytes, etc.) (S3 Fig). All histological assessments were blinded to eliminate observational bias by the assessor of the tissues. As a quality control measure, we re-assessed a subsample of histological sections a second time and compared the resultant maturity values between the initial and the repeat analysis. The calculated mean maturity values obtained for the two analyses differed by <1%.

#### Hematological analyses

Plasma samples were used to measure glucose and vitellogenin concentrations. We chose glucose as a biomarker for the overall metabolic physiology of the organism as it is related to short-term stress and protein metabolism and may also be indicative of malnutrition. A previous study [39] measured 16 blood related biomarkers, including six metabolic bioindicators, in wild-caught fish (including bluegill sunfish) and found only glucose to exhibit patterns that were consistent with exposure history (ash spill). Other blood parameters, such as triglycerides have also been used in previous ecotoxicological studies as indicators of bioenergetic status in fish (for example [66]), but these authors also noted the variability of this endpoint in resident fish. Given the number of fish assessed in the current study, as well as the ease and reliability of glucose measurements, we focused on glucose as an energetic indicator in the current study. Using 1 μL of plasma, a TRUEbalance Blood Glucose Monitor (Moore Medical, Farmington, CT) was used (detection range of 20 to 600 mg/dL) to determine plasma glucose concentrations, a proxy measure for whole body energetics.

Plasma vitellogenin was measured by antibody-capture competitive enzyme-linked immunosorbent assay (ELISA) incorporating a sunfish-validated anti-vitellogenin polyclonal antibody and purified sunfish vitellogenin as standard [67, 68] and followed previously published protocols [4]. R-squared values associated with the standard curve plots were above 0.97, with the majority at 0.99, using at least seven standard concentrations ranging from 4.8 to 0.075 μg/mL. Each plate contained a set of standards for curve generation, and was read precisely at 20 minutes post-TMB addition. The minimum detection limit utilizing this standard curve was 3.75 μg/mL.

Analysis of plasma samples was randomized across ELISA plates and analysis days to minimize assay drift. A high degree of homology within monophyletic taxa has been reported for the vitellogenin gene of at least three monophyletic taxa: the order cypriniformes [69], the genus *Mugil* [70], and the genus *Micropterus* [69]. Given these data, it is likely that the antibody-capture competitive ELISA incorporating a sunfish-validated anti-vitellogenin antibody and purified sunfish vitellogenin as standard would perform well for the four *Lepomis* species in the current study. Four plasma samples from each plate were replicated on a second plate to examine cross-plate variability.

### Data analysis

The richness of the chemical data matrix and its inherent complexity required *a priori* reductive processes to allow for its integration with the equally rich and complex biological matrix (a more complete treatment of the chemical data is reported in the companion manuscript, Elliott et al., in press). Chemical results were reduced to only one concentration for each chemical per site in each analyzed matrix (sediment and water). This was achieved by identifying the maximum concentration that was detected for each chemical at each site, for both water and sediment. The maximum concentration for each site was used for this screening method because the dataset had an abundance of left-censored data with few samples per site, resulting in insufficient data to appropriately use other estimation techniques, such as maximum likelihood. In addition, the maximum concentration for a given chemical within a given matrix also represents the concentration of greatest toxicological concern, which is relevant when evaluating with biological samples.

Finally, the chemical dataset was further reduced by combining chemicals into 15 classes (S2 Table). The classes used for this analysis includes: Alkylphenols, Fecal Indicators, Flame Retardants, Fragrances, Hormones, Industrial, Insect Repellants, Organohalides, Other, Polycyclic Aromatic Hydrocarbons (PAHs), Pesticides, Pharmaceuticals, Phenolics, Plasticizers, and Sterols. To achieve this, chemical concentrations were summed for each class. Total chemical class concentrations were used to evaluate differences between rivers. The chemical analysis provided by the USGS National Water Quality Laboratory by default included several classes of chemicals not usually considered CECs. These included PAHs, phenolics, sterols, and fecal indicators. Despite the focus of this study on CECs, we elected to include these data in the analyses to provide the most comprehensive assessment of explanatory variables possible.

To more broadly assess the estrogenic potential of the water samples, an estradiol equivalency value (EEQ) was calculated for each study site. EEQs were derived based on those reported in literature [71–75]. The sum EEQ was attained by multiplying individual chemical concentrations by the EEQ multiplier (S2 Table), and then summing the results.

Analysis of biological endpoints was conducted in Graphpad Prism V 6.0a (Oxnard, CA). Gonad maturity values were arcsin-transformed prior to analysis [76]. Plasma vitellogenin and plasma glucose concentrations were log_10_-transformed prior to analysis [4]. To allow for statistical analysis of samples below or above the lower and upper detection limits, respectively, the following convention was applied: vitellogenin or glucose concentrations below the lower assay detection limit were recorded as ½ the calculated lower detection limit of the assay (1.88 μg/mL for plasma vitellogenin, 5mg/dL for glucose). Values above the upper detection limit of each assay were recorded as the nominal upper detection limit (8,000 ug/mL and 600 mg/dL, respectively). Normality of data was tested using a D’Agostine and Pearson omnibus normality test. As many data sets were found to lack normality or were data of ordinal nature (hepatocyte vacuolization, sum of pathologies), all data were analyzed using Kruskal-Wallis with Dunn’s post-test. Presence/ absence data for biological endpoints were assessed using a Fisher’s Exact Test.

To examine differences between in-river locations and differences between study years (for Cuyahoga and Raquette rivers only), data from male and female fish were analyzed separately using generalized linear mixed models and the GLIMMIX procedure in SAS V9.3 (SAS Institute, Inc., Cary, NC). In the primary analysis, differences between in-river locations (downstream, middle or upstream) were tested using a mixed model in which the collection/caging site was the fixed effect and river and sampling locations within years were random effects. The data from the Raquette and Cuyahoga rivers were used in a secondary analysis to test whether there were differences between fish captured in 2013 and 2014. In these models, year, collection/caging site, and their interaction were included as fixed effects while river and sampling location were considered random effects.

Next, we conducted the canonical redundancy analysis (RDA) on the data matrix using the vegan package in R [77] and obtained a new set of multivariate traits (see S1 File for R code developed for the current study). These multivariate axes remove the effects of correlations between closely aligned variables. The RDA technique was used to relate the matrix of biological response variables (Y) to the matrix of chemical explanatory variables (X; sums of concentrations by class). Explanatory variables were log-transformed to remove considerable skewness in the distributions. At some sites, certain chemical classes contained only censored (non-detect) data. In these cases, we substituted one-half the lowest sum of concentrations for the chemicals that were detected among the remaining sites prior to log-transformation. Although simple substitution techniques may impart bias, the rank order of sums by chemical class was preserved, while allowing the ability to explore general patterns in the data as revealed by the RDA. More roust techniques to estimate censored data, such as maximum likelihood estimation, require more samples than were collected for this study.

A secondary RDA was performed using only pharmaceutical results in water samples as explanatory variables (X, sum of concentrations by subclass). This secondary RDA was performed because the class Pharmaceuticals includes 119 chemicals (S2 Table), and the classification is mostly arbitrary with no common mode-of-action. Their only common feature is their pharmacological origin. Subclasses of pharmaceuticals were classified based on common mode-of-action. Only subclasses with a detection in at least one sample and those that have the potential to impact the biological endpoints measured were used for analyses. Subclasses included: anesthetics, anticonvulsants, antidepressants, antidiabetics, antihistamines, antimicrobials, cardiovascular drugs, muscle relaxants, nonsteroidal anti-inflammatories (NSAID), and opioids (S2 Table).

## Results

### Water and sediment chemistry and estradiol equivalency values (EEQs)

Contaminants of emerging concern were ubiquitous in water and sediment samples collected from all sites [43, 44]. A detailed analysis of water and sediment chemistry is provided in the companion manuscript (Elliott et al., in review). All 15 classes of CECs (S2 Table) were detected in at least one site for both water (Table 2) and sediment (Table 3) samples, although the number of CEC classes detected per site in water samples was always larger than in sediment (mean 12.9 classes vs. 8.9 classes). Insect repellents, sterols and several other chemicals were documented in water samples from all 27 field sites (Table 2), while pharmaceuticals and pesticides were detected in all sites but one. Fecal indicators and PAHs were the most frequently detected classes in sediment samples (Table 3).

**Table 2:** CEC concentration (μg/L) in water summed by class.

River	Site	Alkylphenols	Fecal indicators	Flame retardants	Fragrances	Hormones	Industrial	Insect repellants	Organohalides	Other	PAHs	Pesticides	Pharmaceuticals	Phenolics	Plasticizers	Sterols	Total conc.	CEC classes with detections (#)	Sum EEQ
**Fox River**	LBM-1	0.4360	0.0122	0.0000	0.0000	0.0009	0.0257	0.0095	0.0000	0.0419	0.7976	0.1366	0.0350	0.0000	0.0000	8.6115	10.1069	10	0.0018
	LLB-3	0.8010	0.0037	0.5753	0.0657	0.0018	0.7575	0.4140	0.1912	0.1039	0.0156	0.7248	1.3723	0.0086	0.0000	7.4794	12.5147	15	0.0030
	LLB-5	0.1630	0.0092	0.4038	0.0073	0.0010	0.1332	0.0531	0.0000	0.1587	0.0609	0.1768	0.3507	0.0113	0.0000	16.5389	18.0679	14	0.0027
	LLB-6	0.4030	0.0115	0.4048	0.0113	0.0027	0.1544	0.0759	0.2664	0.1337	0.1053	0.1658	0.3772	0.0107	0.0000	37.0484	39.1711	15	0.0042
	FXR-13	0.0441	0.0105	0.3387	0.0134	0.0000	0.1514	0.0526	0.0289	2.8949	0.0263	0.1392	1.2433	0.0126	0.0000	30.0215	34.9774	13	0.0009
**Chicago River**	CHI-112	2.0860	0.0883	1.5552	0.3890	0.0344	1.2896	0.0710	1.0688	13.7675	1.7306	0.4617	6.9086	0.0519	0.0000	175.8126	205.3152	15	0.0273
	CHI-RP4	3.7970	0.0839	3.7952	1.5960	0.0179	3.3733	0.2010	0.8572	1.7114	0.1713	0.6912	19.3561	0.0316	0.0000	55.8882	91.5712	15	0.0199
	CHI-36	4.1020	0.0948	3.2362	1.4398	0.0155	4.0191	0.2450	0.8027	1.8917	0.2478	0.7791	29.5604	0.0472	0.0000	58.7888	105.2701	15	0.0176
**Little Calumet River**	CHI-56	0.1080	0.0181	0.5735	0.0986	0.0011	0.4299	0.0523	0.0934	0.3306	0.3694	0.3282	0.9179	0.0000	0.0000	4.9967	8.3176	14	0.0025
	CHI-RP7	3.5620	0.0298	1.7397	0.9712	0.0107	8.3171	0.1730	0.4447	1.7066	0.2372	1.4867	15.4190	0.0508	0.5512	28.9280	63.6278	15	0.0164
	CHI-76	3.5220	0.0335	1.6229	1.0254	0.0163	3.2037	0.2550	0.2297	1.5475	0.4103	2.1302	14.1579	0.0878	0.3860	28.2055	56.8337	15	0.0204
**Clinton River**	CLI-5	0.0000	0.0120	0.2890	0.0000	0.0000	0.0300	0.0663	0.0134	0.2100	0.2403	0.0493	0.0978	0.0000	0.0000	2.2516	3.2596	10	0.0005
	CLI-4	0.0000	0.0000	0.0000	0.0000	0.0009	0.0000	0.0244	0.0212	0.4756	0.0000	0.0354	0.0000	0.0000	0.0000	8.8595	9.4169	6	0.0014
	CLI-3	0.0000	0.0000	0.3674	0.1160	0.0000	0.2609	0.0616	0.0389	0.2021	0.1220	0.0632	0.9916	0.0000	0.0000	1.9371	4.1607	10	0.0005
	CLI-2	0.0000	0.0100	0.4953	0.0771	0.0000	0.6075	0.1470	0.0167	0.8905	0.7770	1.1383	0.7533	0.0000	0.0000	3.1026	8.0152	11	0.0005
	CLI-1	0.0000	0.0037	0.5110	0.1319	0.0007	0.6447	0.1340	0.0126	0.4723	0.2913	0.9627	0.6481	0.0110	0.0000	2.2636	6.0874	14	0.0013
**Cuyahoga River**	CUY-5	0.0000	0.0000	0.0000	0.0061	0.0008	0.0097	0.0450	0.0000	0.5063	0.0749	0.0717	0.0440	0.0000	0.0000	7.0392	7.7977	9	0.0010
	CUY-4	0.0000	0.0064	0.9773	0.0872	0.0006	0.7697	0.0754	0.0204	0.5740	0.5783	0.6677	0.8872	0.0135	0.0000	5.5022	10.1598	14	0.0198
	CUY-3	2.8480	0.0265	2.6702	0.6660	0.0122	2.4792	0.3300	0.6967	1.5919	0.3400	0.5461	41.6409	0.1400	0.3070	19.9814	74.2759	15	0.0077
	CUY-2	0.1940	0.0056	1.3378	0.2249	0.0009	1.2236	0.1560	0.3270	1.0038	1.4498	0.6745	7.0313	0.0138	0.0000	6.1510	19.7940	15	0.0029
	CUY-1	1.2335	0.0060	1.0889	0.1590	0.0025	0.8574	0.1240	0.0895	0.5492	0.4024	0.7776	4.4240	0.0150	0.0117	9.2620	19.0026	15	0.0041
**Raquette River**	RAQ-6	0.3340	0.0048	0.0000	0.0000	0.0004	0.0000	0.0758	0.0000	0.0541	0.0000	0.0073	0.0292	0.0121	0.0000	3.8308	4.3484	9	0.0013
	RAQ-5	0.6238	0.0307	0.3894	0.0745	0.0048	0.7607	0.1030	0.1430	0.9026	0.0055	0.0014	1.4647	0.0119	0.0000	4.9694	9.4854	14	0.0035
	RAQ-4	0.7861	0.0066	0.1294	0.0000	0.0084	0.0000	0.1520	0.0200	5.3461	0.0000	0.0079	0.4993	0.0183	0.0000	2.3801	9.3542	11	0.0082
	RAQ-3	1.1971	0.0032	0.0448	0.0000	0.0014	0.0000	0.0063	0.0159	0.3955	0.0105	0.0000	0.0427	0.0000	0.0000	2.2315	3.9489	10	0.0094
	RAQ-2	0.6281	0.0033	0.2450	0.0000	0.0027	1.1625	0.0248	0.1500	0.1960	0.1249	0.0046	0.0612	0.0125	0.0000	2.2128	4.8284	14	0.0280
	RAQ-1	0.7003	0.0065	1.3304	0.0604	0.0000	8.8684	0.0741	0.0100	0.1128	0.0120	0.0602	1.1179	0.0118	0.0000	1.8829	14.2478	14	0.0011

Sum of chemical concentrations (μg/L) per chemical class for each site in water samples collected in 2013 and 2014, Total conc., sum of all chemical classes for each site (μg/L), number of CEC classes detected for each site, and sum EEQ (μg/L) for each site (see S2 Table with list of chemicals in each class). 0.0000 = not detected.

**Table 3:** CEC concentration in sediment summed by class.

River	Site	Alkylphenols	Fecal indicators	Flame retardants	Fragrances	Hormones	Industrial	Insect repellants	Organohalides	Other	PAHs	Pesticides	Pharmaceuticals	Phenolics	Plasticizers	Sterols	Total conc.	CEC classes with detections (#)	Sum EEQ
**Fox River**	LBM-1	0	1371	0	0	10	127	0	4	58	3924	6	22	961	0	2090	8573	8	3.07
	LLB-3	28	32	37	26	0	7	0	0	1	111	0	18	0	0	0	260	8	0.17
	LLB-5	0	823	0	4	8	276	0	8	124	4869	0	0	664	45	18090	24911	10	18.59
	LLB-6	0	290	0	0	3	318	0	0	153	5918	0	0	353	93	0	7129	7	1.97
	FXR-13																		
**Chicago River**	CHI-112	18	77	111	45	19	276	0	9	326	4126	0	52	278	54	0	5391	12	7.15
	CHI-RP4	19	62	107	364	5	24	18	32	23	129	47	371	179	0	0	1380	13	2.03
	CHI-36	845	691	105	738	9	612	0	61	159	1651	57	200	830	0	7470	13429	13	3.66
**Little Calumet River**	CHI-56	47	138	154	118	3	448	0	55	341	4178	0	119	911	136	0	6649	12	2.25
	CHI-RP7	3242	44	159	673	10	1960	0	1160	1106	13369	2	309	1010	167	4010	27221	13	12.93
	CHI-76	3756	108	94	702	21	502	35	372	654	4458	0	170	4480	380	0	15732	13	25.86
**Clinton River**	CLI-5	0	44	0	0	0	0	0	0	4	79	0	0	0	0	0	127	3	0.00
	CLI-4	0	169	0	0	1	0	0	0	10	14	0	0	15	0	7130	7340	6	7.93
	CLI-3	23	125	49	208	1	899	0	0	502	43640	0	3	55	65	1680	47250	11	0.74
	CLI-2	1261	313	46	93	0	514	0	31	205	6911	0	0	203	136	2220	11935	12	4.96
	CLI-1	47	272	0	115	1	702	0	0	361	8920	0	0	136	171	16360	27086	10	12.73
**Cuyahoga River**	CUY-5	0	50	0	0	0	0	0	0	3	65	0	1	11	0	0	129	4	0.00
	CUY-4	24	138	0	44	2	421	0	0	209	7160	0	118	102	45	4300	12563	11	0.55
	CUY-3	14	43	30	104	0	63	0	45	28	1006	0	95	20	0	0	1448	11	0.02
	CUY-2	10	136	17	51	1	172	0	0	69	3844	0	81	95	26	2240	6742	12	0.08
	CUY-1	14	178	0	56	1	137	0	0	46	1634	0	88	59	22	2400	4635	11	0.64
**Raquette River**	RAQ-6	0	25	0	0	0	0	0	0	4	157	0	0	0	0	0	186	4	0.03
	RAQ-5	0	7	0	0	0	0	0	0	2	88	0	0	0	0	0	97	3	0.00
	RAQ-4	0	24	0	0	0	0	2	0	0	0	0	0	12	7	0	46	4	0.02
	RAQ-3	0	165	117	0	2	212	0	16	104	4968	0	12	72	0	0	5668	8	0.29
	RAQ-2	0	212	0	0	3	66	0	0	377	844	0	0	58	0	11760	13319	7	11.76
	RAQ-1	0	73	0	0	0	43	0	0	11	929	0	0	16	17	0	1090	7	0.04

Sum of chemical concentrations (μg/kg) per chemical class for each site in sediment samples collected in 2013 and 2014, Total conc., sum of all chemical classes for each site (μg/kg), number of CEC classes detected for each site, and sum EEQ (μg/kg) for each site (see S2 Table with list of chemicals in each class). 0.0000 = not detected.

The highest total chemical concentrations in water samples were detected in the Chicago River, all but the most upstream site of the Little Calumet River, and site CUY-3, which is located immediately downstream of a municipal wastewater treatment plant in the Cuyahoga River (S4 Fig). These sites also had the largest number of chemicals detected in water samples (S4 Fig). The sites in the Clinton River consistently had lower concentrations and fewer chemicals detected in water samples than the other rivers. For most rivers, the most upstream site contained the lowest concentration and fewest number of chemicals detected in water samples. Similar to water samples, the Chicago and Little Calumet Rivers had the largest number of chemicals detected at each site for sediment samples (S5 Fig). However, a Clinton River site (CLI-3) had the highest total chemical concentration (S5 Fig), with almost twice as high of a concentration (45,570 μg/kg versus 23,211 μg/kg) as the next highest site (CHI-RP7 in the Little Calumet River).

In water samples, the sum estradiol equivalency (EEQ, Table 2) was the highest at one site in the Raquette River (RAQ-2: 0.028 μg/L EEQ), followed closely by a site in the Chicago River (CHI-112: 0.027 μg/L EEQ). The Chicago and Little Calumet Rivers had higher average EEQ values (0.021 μg/L and 0.013 μg/L, respectively) as compared to the other rivers. The Chicago River had decreasing aqueous EEQ values with distance downstream, while the Little Calumet and Raquette Rivers had increasing EEQ values with distance downstream. The lowest concentrations were recorded at three of the five sites in the Clinton River (CLI-2, CLI-3, CLI-5: 0.0005 μg/L EEQ) For sediment samples, there were fewer noticeable patterns in EEQ values (Table 3). The Little Calumet River did show an increase in sediment EEQ values in a downstream direction, which was congruent with EEQ values in water samples. The highest EEQ value in sediment was detected in the Little Calumet River (CHI-76: 25.86 μg/kg EEQ), followed by the Fox River (LLB-5: 18.59 μg/kg EEQ). The lowest EEQ values (all 0.0000 μg/kg EEQ) were detected in upstream sites in the Clinton River (CLI-5), Cuyahoga River (CUY-5) and Raquette River (RAQ-5). The Little Calumet River had the highest average EEQ value (13.68 μg/kg) as compared to the other rivers. It is interesting to note that while the RAQ-2 site is not the highest value as compared to other sites (11.76 μg/kg), it is much higher than any of the other sites in the Raquette River (next highest was at RAQ-3: 0.29 μg/kg)

### Biological endpoints

**Fig 2:**
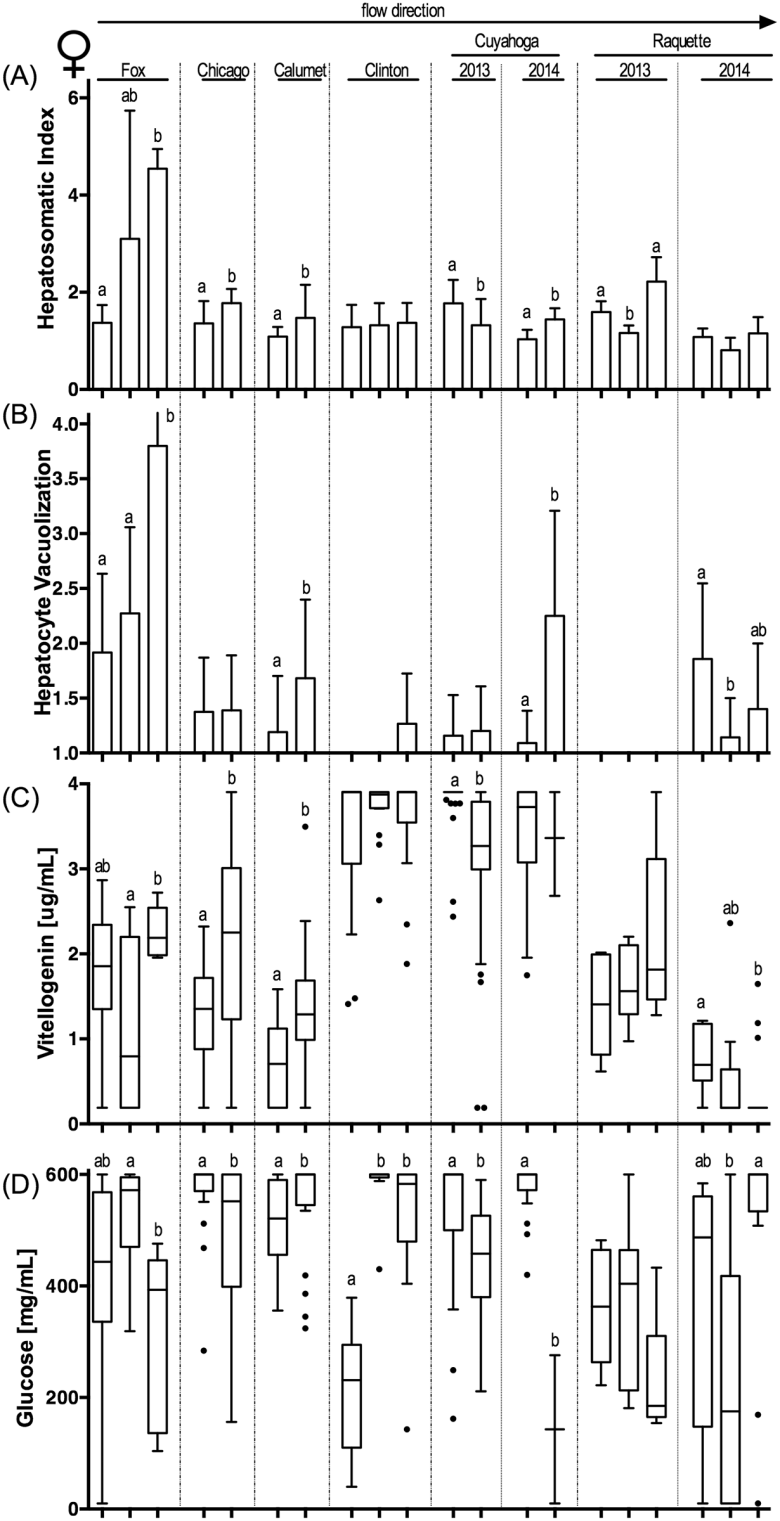
Biological endpoints in resident female sunfish. (A) hepatosomatic index; (B) prevalence of vacuoles in hepatocytes ranked on a severity scale of 1 to 4; (C) plasma vitellogenin concentration (μg/mL); and (D) plasma glucose concentration (mg/mL). Sample river location located above panels (A) and (B), with columns representing upstream to downstream within each river from left to right. Specific sample site identification can be found in Table 1. Column graphs indicate mean + standard deviation in panels (A) and (B). Box-and-whisker plots indicate range, 25^th^ and 75^th^ percentiles, and mean values in panels (C) and (D). Statistical significance (Kruskal-Wallis with Dunn’s post-test; p<0.05) within panels are identified by letters. P-values are summarized in S4 Table. Sample size provided in S3 Table.

**Fig 3:**
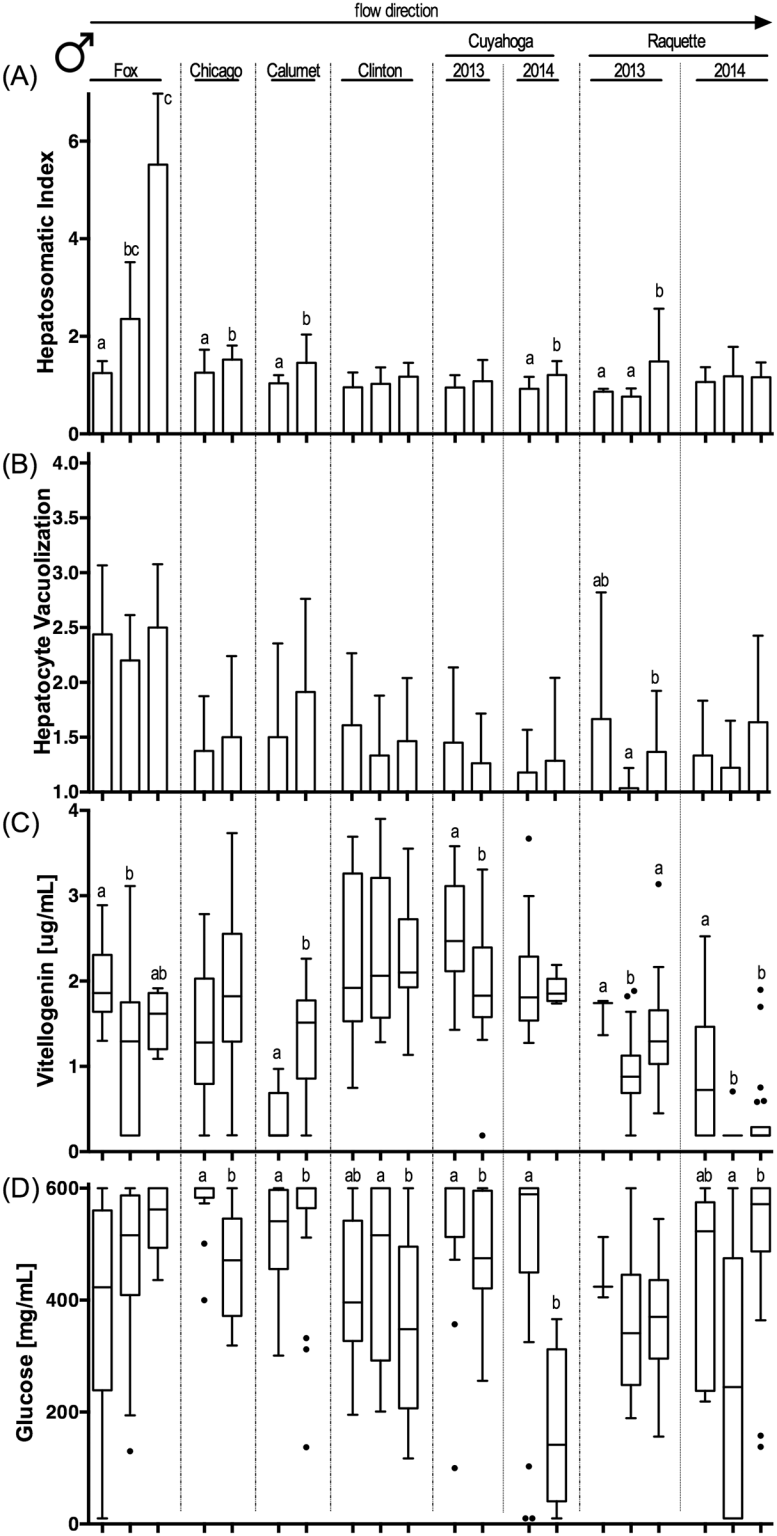
Biological endpoints in resident male sunfish. (A) hepatosomatic index; (B) prevalence of vacuoles in hepatocytes ranked on a severity scale of 1 to 4; (C) plasma vitellogenin concentration (μg/mL); and (D) plasma glucose concentration (mg/mL). Sample river location located above panels (A) and (B), with columns representing upstream to downstream within each river from left to right. Specific sample site identification can be found in Table 1. Column graphs indicate mean + standard deviation in panels (A) and (B). Box-and-whisker plots indicate range, 25^th^ and 75^th^ percentiles, and mean values in panels (C) and (D). Statistical significance (Kruskal-Wallis with Dunn’s post-test; p<0.05) within panels are identified by letters. P-values are summarized in S4 Table. Sample size provided in S3 Table.

**Fig 4:**
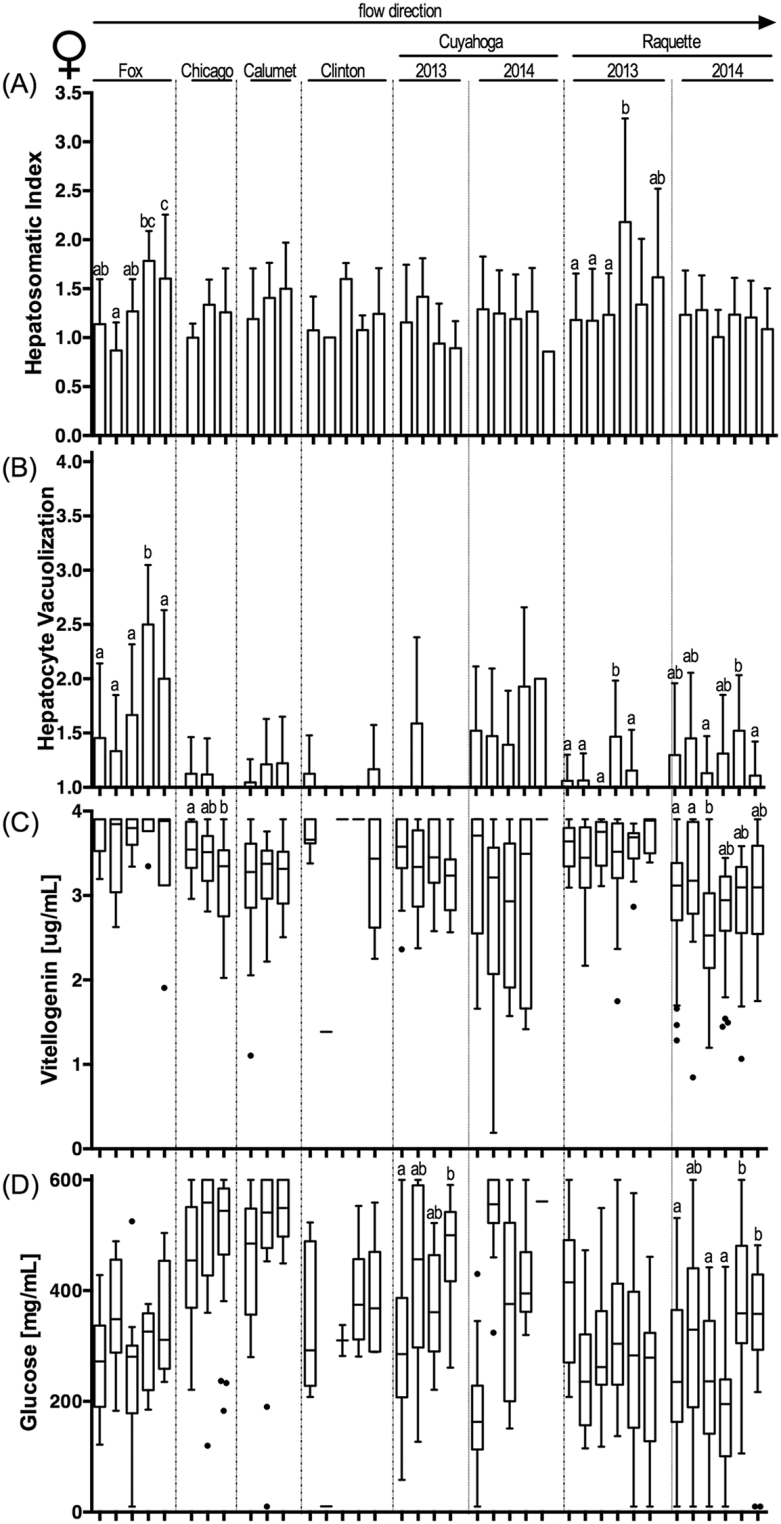
Biological endpoints in caged female sunfish. (A) hepatosomatic index; (B) prevalence of vacuoles in hepatocytes ranked on a severity scale of 1 to 4; (C) plasma vitellogenin concentration (μg/mL); and (D) plasma glucose concentration (mg/mL). Sample river location located above panels (A) and (B), with columns representing upstream to downstream within each river from left to right. Specific sample site identification can be found in Table 1. Column graphs indicate mean + standard deviation in panels (A) and (B). Box-and-whisker plots indicate range, 25^th^ and 75^th^ percentiles, and mean values in panels (C) and (D). Statistical significance (Kruskal-Wallis with Dunn’s post-test; p<0.05) within panels are identified by letters. P-values are summarized in S4 Table. Sample size provided in S3 Table.

**Fig 5:**
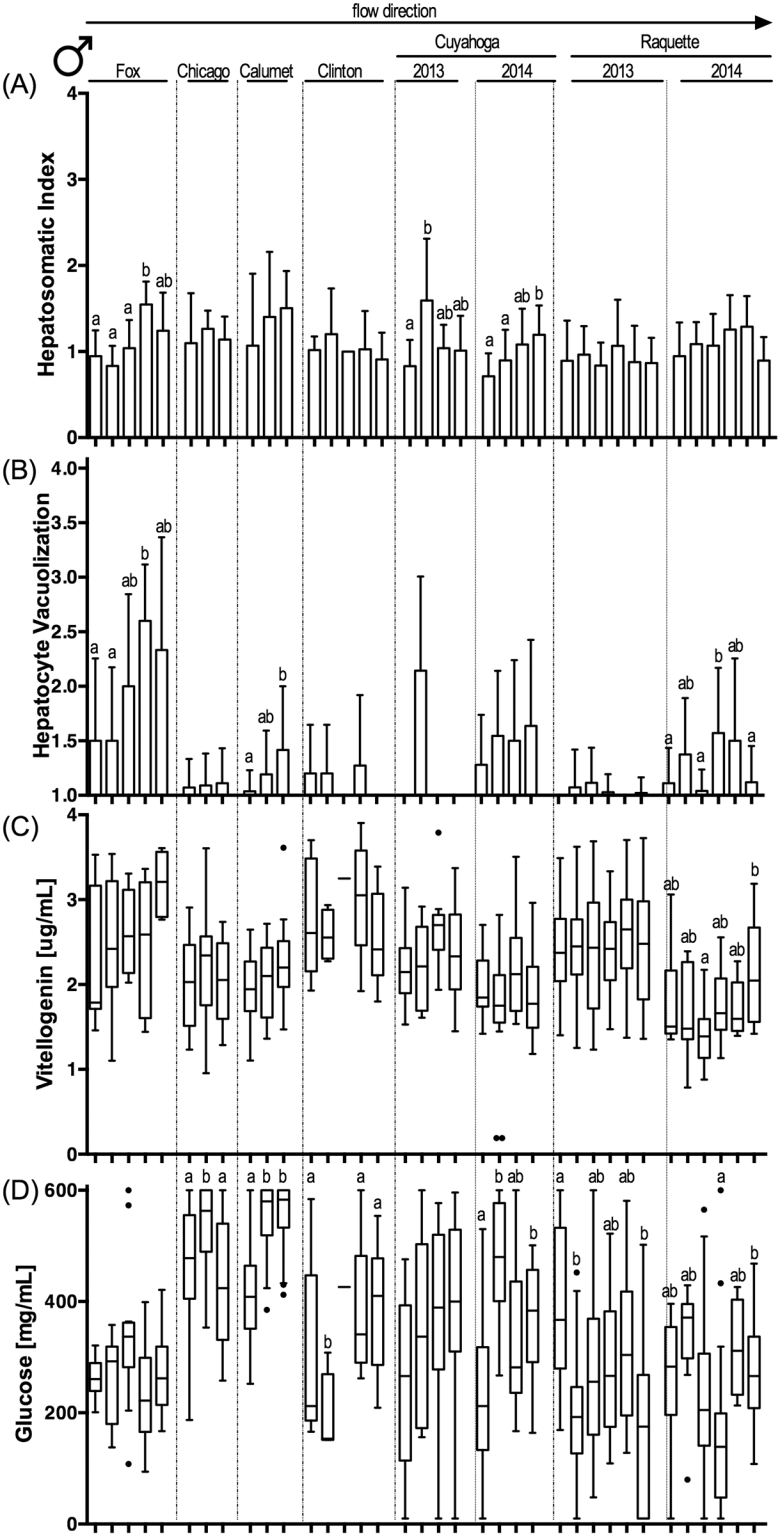
Biological endpoints in caged male sunfish. (A) hepatosomatic index; (B) prevalence of vacuoles in hepatocytes ranked on a severity scale of 1 to 4; (C) plasma vitellogenin concentration (μg/mL); and (D) plasma glucose concentration (mg/mL). Sample river location located above panels (A) and (B), with columns representing upstream to downstream within each river from left to right. Specific sample site identification can be found in Table 1. Column graphs indicate mean + standard deviation in panels (A) and (B). Box-and-whisker plots indicate range, 25^th^ and 75^th^ percentiles, and mean values in panels (C) and (D). Statistical significance (Kruskal-Wallis with Dunn’s post-test; p<0.05) within panels are identified by letters. P-values are summarized in S4 Table. Sample size provided in S3 Table.

Biological endpoints (see S2 File for all raw biological data) associated with exposure to CECs exhibited a complex pattern across sites and whether resident or caged fish were assessed (Table 4; Figs 2–5; S6–S9 Figs; for sample size see S3 Table). Our biological sampling design allowed for a nominal total of 224 statistical analyses (eight river sampling events (six rivers plus two years of sampling in two rivers) x two sexes x two fish sources (resident/caged) x seven endpoints (excluding pathologies)). Some data gaps reduced this number to an actual 217 statistical comparisons of biological effects by river, of which 82 yielded statistically significant differences among sites in a river. Overall, 56 significant differences in endpoint expression were identified in resident fish, while only 26 significant differences were present in caged fish (Fisher’s exact test, p<0.0001). The frequency of observed effects did not vary significantly between the sexes (Fisher’s Exact Test, p>0.05; 30 effects in resident females, 26 in resident male fish; 9 and 17 for caged female and male fish, respectively). Plasma glucose concentrations differed significantly most frequently among fish collected from different sites or reaches within a river (21 instances by sex and resident vs. caged fish), followed by HSI (16 differences), plasma vitellogenin concentrations (13 differences) and prominence of hepatocyte vacuoles (11 differences). It is noteworthy that CF, GSI and gonad maturity varied less frequently among sites (8, 6, 7 differences, respectively) and that most of the variability was observed among resident fish. Caged fish exhibited little variability in these endpoints, likely as a reflection of the homogeneity of hatchery-reared caged fish that were acquired at a similar age and reproductive status.

**Table 4:** Biological responses.

Sex	Fish	CF	GSI	HSI	Log_10_Vtg	Glucose (mg/ml)	Maturity	Hepatocyte vacuolization	Sum of pathologies
Male	Resident (n = 15)	1.78±0.4 0.59–2.2	0.61±0.3 0.25–1.27	1.53±1.2 0.92–5.12	1.56±0.6 0.42–2.33	458±72 334–577	1.75±0.5 1.04–2.51	1.63±0.4 1.09–2.50	1.21±0.3 0.58–1.88
	Caged (n = 27)	1.58±0.2 1.25–2	1.09±0.5 0.40–2.09	1.08±0.2 0.77–1.55	2.35±0.4 1.78–3.25	348±108 192–555	2.19±0.4 1.10–3.03	1.34±0.4 1.00–2.60	1.13±0.4 0–2.0
Female	Resident (n = 15)	1.71±0.4 0.59–2.1	3.51±2.6 0.98–10.06	1.74±0.9 0.95–4.54	2.05±1.2 0.69–3.71	454±112 218–585	2.59±0.5 2.09–3.78	1.54±0.7 1.00–3.80	1.20±0.4 0.57–1.8
	Caged (n = 27)	1.58±0.2 1.41–2	4.45±2.5 2.27–11.47	1.25±0.2 0.87–1.78	3.27±0.5 1.39–3.9	358±119 10–547	3.08±0.3 2.74–4	1.30±0.3 1–2.5	1.07±0.4 0.5–2

Mean measured biological responses (± standard deviation) and range of values of resident and caged female and male sunfish sampled in 2013 and 2014. Biological responses include condition factor (CF), gonadosomatic index (GSI), hepatosomatic index (HSI), Log_10_ plasma vitellogenin concentration (Log_10_Vtg), plasma glucose concentration, maturity, hepatocyte vacuolization, and sum of pathologies.

When statistical analyses for all data sets were compared (both ANOVA and generalized linear mixed model), few patterns were found to be consistent across fish sex and/or resident and caged fish. Only in the Fox River were several biological endpoints consistently altered following a consistent upstream to downstream pattern. Here, CF decreased significantly from upstream to downstream reaches for resident male and female fish (Figure A in S6 Fig, Figure A in S7 Fig). This decrease was counter-directional to a consistent increase in HSI and liver hepatocyte vacuolization for both resident and caged fish (except vacuole prominence in resident male fish, Figs 2–5, S4 Table). Interestingly, plasma glucose concentrations showed significant but opposing changes in the Fox River resident female (Figure D in Fig 2, S4 Table) and male fish (Figure D in Fig 3, S4 Table), with females having decreasing plasma glucose concentrations and males increasing concentrations with distance downstream. These resident fish results were not paralleled by findings in caged fish from the Fox River.

Fish sampled in other rivers exhibited fewer significant differences in endpoint expression between sites or reaches (Chicago, Little Calumet, Clinton, Cuyahoga 2014) or differences were inconsistent between sites, sexes, and resident or caged fish (Cuyahoga 2013, Raquette 2013). It is noteworthy that in rivers that were sampled twice (Cuyahoga, Raquette), patterns were not consistent between years. Using a generalized linear model, significant differences for CF (p = 0.016) and maturity (p = 0.0016) were found between years for resident male fish (independent of river). Significant differences between years (independent of river) were also found for GSI and maturity in caged female and male fish (p<0.001 for all). In contrast, river location (upstream, middle, downstream reach) was a poor indicator to predict the expression pattern of any endpoint in female or male, resident or caged fish.

### Canonical redundancy analysis

**Fig 6:**
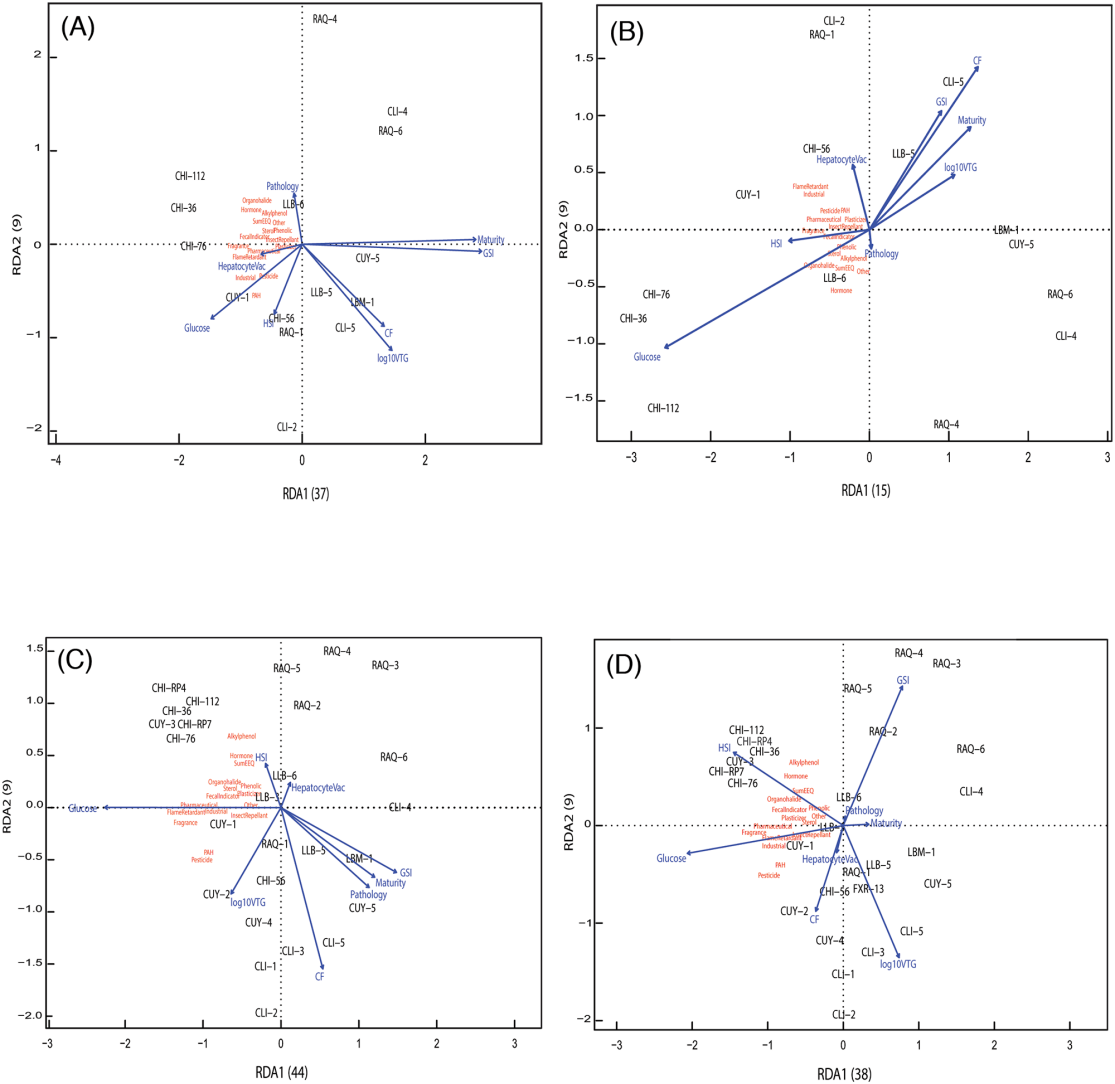
Canonical redundancy analysis for water samples and biological results. (A) Resident females, (B) Resident males, (C) Caged females, and (D) Caged males. Number in parentheses on axes indicate the percent variability that is explained by that axis. Sample site information can be found in Table 1. Sample class information can be found in Table 2. Biological response information can be found in Table 4, Figs 2–5, S6–S9 Figs, and S3 Table.

The first two axes of the RDA express a mean 43.5% of the variance (34.5% and 9.0% for the first (X) and second (Y) axis, respectively) across both sample matrices (water and sediment) and all fish groupings (resident/caged, female/male; Fig 6; S10 and S11 Figs). The explanatory power of the analyses differed little if biological endpoints were compared only against water, sediment or both matrices combined (43.1%; 46.4%; 41.0%, respectively). Among the fish groupings, only resident male fish produced substantially lower first and second axis values (sum mean: 29.2%) when compared to resident female or caged female or male fish (48.6%; 49.2%; 47.2%, respectively). This is consistent with the partitioning of constrained and unconstrained variance of the data set (S5 Table). When constrained variance is greater than unconstrained variance in an RDA analysis, then the results indicate that much of the variation in the response variables (biological data set) is related to the explanatory variables (chemical data set). As a result, more of the variability in biological data in all female and caged male fish is due to the explanatory variables while the origin for the biological variability of resident male sunfish is less certain (Fig 6; S10 and S11 Figs).

In caged female fish, ordination identified pharmaceuticals (p<0.001) and PAHs (p = 0.008) as the significant explanatory variables in water samples, while fragrances were the significant explanatory variable for caged males (p<0.001). In sediment samples, RDA identified sum EEQ (p = 0.035) for resident females, and fragrances for caged females and males (p<0.001 for both) as significant explanatory variables. When sediment and water were combined, ordination identified pharmaceuticals and PAHs for both caged females (p<0.001 and p = 0.006, respectively) and males (p<0.001 and p = 0.008, respectively).

The RDA identified consistent patterns of endpoint expression (response variable) and chemical exposure (explanatory variable) for resident and caged fish and for both sexes (Fig 6; S10 and S11 Figs). The vector length for each biological response variable indicates the strength of the relationship between the response variable the explanatory variables. Vectors whose angle approaches 90° have little correlation with each other; vectors approaching 0° are strongly correlated and vectors approaching 180° are negatively correlated. Plasma glucose concentrations are consistently driving the differences along the first axis of the RDA plots and are consistently negatively correlated by GSI and gonad maturity (Fig 6; S10 and S11 Figs) suggesting a strong negative correlation between the former and two latter biomarkers. In contrast, CF aligns to the second axis of most RDA plots while HSI and the prominence of hepatocyte vacuoles are commonly aligned with each other. In caged fish, the association between HSI and prominence of hepatocyte vacuoles is weaker than in resident fish. Plasma vitellogenin concentrations often correlated with GSI and maturity in resident sunfish along the first RDA axis but are more commonly associated with the second RDA axis for caged sunfish. The alignment of pathologies with other biological endpoints is variable and explains little of the differences in the data matrices except in the analysis of sediment chemistry (S10 Fig). When examining endpoint expression in the context of CEC classes, plasma glucose appears to align more closely with urban-associated CECs (e.g., flame retardants and plasticizers), while HSI and prominence of hepatocyte vacuoles align more closely with endocrine active CECs (EEQ, steroid hormones) and are opposed by CF and often maturity.

The results of the pharmaceutical subclass RDA paralleled those observed in the other RDAs (S12 Fig). Resident male fish produced substantially lower first and second axis values (22.3%) when compared to resident female or caged female or male fish (52.1%; 58.3%; 52.6%, respectively). In addition, this observation is consistent with the partitioning of the constrained and unconstrained variance of the dataset (S5 Table). For biological responses, glucose continued to be the most important driver of the results and was negatively correlated with CF, GSI, and maturity. Hepatocyte vacuolization and HSI often correlated along the second axis. In the redundancy analysis, no pharmaceutical subclasses were identified as significant explanatory variables in resident females (all p>0.05), while only anesthetics were identified for caged females (p<0.001) and opioids for caged males (p<0.001).

## Discussion

We applied two lines of evidence gathered from resident and caged sunfish to assess the biological effects of CEC occurrence on fish in Laurentian Great Lakes tributaries. First, we assessed resident and caged fish for biological effects commonly associated with CEC exposure. Second, we evaluated whether the measured biological effects in fish were associated with the concentration of CECs in the surrounding aquatic environment.

### CEC presence in Great Lakes tributaries

Contaminants of Emerging Concern were detected in all water and sediment samples collected across the Great Lakes tributaries. CEC presence extended to two putative reference sites we identified *a priori* based on their limited shoreline development and upstream locations. This omnipresence was expected as the targeted rivers are known to be subject to anthropogenic impacts. Consequently, the results of the current study are congruent with published reports of ubiquitous CEC presence in water samples from aquatic ecosystems in North America [78, 79], South America [80], Europe [80] (reviewed in [81, 82]), Asia (reviewed in [83]) and Australia [84] leaving little doubt that CECs are a global pollution concern with uncertain consequences for exposed aquatic life.

The results of our water analysis corroborated our predictions that CECs would be most common and at the highest concentrations downstream of municipal wastewater treatment plant outfalls and in effluent dominated rivers. CUY-3, just downstream of an effluent outfall registered some of the highest total contaminant concentration and was matched by most sites in the effluent-dominated Chicago and Little Calumet Rivers. Increasing total CEC concentrations were paralleled with increasing EEQ values, suggesting that estrogenic CECs contribute substantially to the overall CEC concentrations measured. Conversely, upstream sites usually contained fewer CECs and at lower concentrations with less EEQ activity. However, the ubiquitous presence of CECs across all sampling sites may affect study designs as it calls into question the very concept of reference sites. We had identified two putative reference sites (a mostly forested site in the Cuyahoga River and an upstream segment of the Raquette River in the Adirondack Mountains) and already concluded that no reference sites of suitable characteristics existed in the other four rivers. However, even our reference sites were found to contain CECs. Recently published data assessing CEC presence in 50 Minnesota lakes [85] found only three of 50 sampled lakes to be free of CECs. Several lakes without shoreline development, and in one instance within the Minnesota Boundary Waters Canoe Area (an area with strictly limited human access and prohibition of any use of combustion engines), contained detectable concentrations of multiple pharmaceuticals and even illicit drugs. A companion study of 50 Minnesota rivers found only one site free of CECs [86]. Given the population density and agricultural and urban land use intensity around the US portion of the Great Lakes watershed, reference sites practically do not exist. The use of a RDA analysis rectifies the lack of reference sites partially as it determines the degree to which explanatory variables in the data matrix may account for variability in the response variables (biomarkers). The results of a RDA, therefore, represent a “standard curve” against which other sites (for example hypothetical reference sites with little to no CEC contamination) could be extrapolated. Future studies should take the paucity of reference into consideration when identifying study locations and analysis tools.

Similar to other studies [87–90], the site-by-site composition of CECs varied, highlighting the complexity of pollutant source, transport and fate across the Great Lakes, as discussed in greater detail in the companion manuscript (Elliott et al., in review). Despite the large chemical data matrix generated in this study, assessing the full complexity of CEC occurrence and concentration variability remains elusive as these compounds vary dramatically over time. For example, Martinovic et al. [89, 90] demonstrated in two studies order-of-magnitude changes in estrogenic activity in effluent discharged from several treatment facilities in the Upper Midwest. In one of these studies [90] the authors also identified highly variable biological activity associated with CEC presence in storm water runoff in an urban environment. The size of the data matrix in the current study to some extent compensates for the lack of temporal sampling (as it likely captured both high and low CEC occurrence across sites), but temporal CEC variability remains a concern for the interpretation of most environmentally derived biological data.

The three most urbanized rivers in our data set, the Chicago, Little Calumet, and Cuyahoga Rivers predictably contained the highest chemical concentrations, largest number of chemicals detected, and largest diversity of chemical classes in water. Previous studies have also noted the presence of a large number of CECs in highly urbanized aquatic ecosystems [4, 64, 90, 91, 92], particularly those with wastewater treatment plant effluents present. Wastewater treatment plant effluents are a well-studied and consistent source of CECs (reviewed in [93]) and were a prominent source in the current study. For all three rivers, pharmaceuticals factored prominently into the total CEC concentration (S4 Fig), following observations from other studies that detected higher concentrations of pharmaceuticals just downstream of major wastewater outfalls [79, 94]. One caveat to this finding is that the pharmaceutical class encompasses a large number of diverse compounds which may contribute to its common presence.

In contrast to the highly urbanized rivers, the Raquette River had lower chemical diversity, but recorded the highest total EEQ value. While the Raquette River has a larger percent forested land than the other sites, it nevertheless contains multiple municipalities along the river that input CECs via their wastewater treatment facilities and also encompasses dairy farms in its watershed, which may have contributed to the high EEQ values. The differences in chemical diversity between rivers receiving wastewater may be due to differences in the composition of the raw influent [79] and its treatment [95] (reviewed in [96]).

### Biological observations

The variability in biological effects paralleled and in some instances exceeded the complexity observed in the CEC occurrence matrix. While biological endpoints frequently differed between sites and reaches in the same river, the pattern of effect occurrence and the congruence between biological effects was not always intuitive and did not follow the a-priori prediction of greater biological effects consistent with increasing CEC exposure at downstream sites. However, some broad patterns were noted between sampling sites, fish collection methods, and sex. Effects were more common in resident fish than in caged fish. Direct comparisons of biological responses between resident and caged fish are infrequent in the published literature (however, see [58, 97] for examples) but can be informative. Several factors may have contributed to the less pronounced consequences of CEC exposure in caged fish. First, caged fish are only exposed to the site-specific water for two weeks vs. potentially a lifetime for resident fish. In a previous study, Burki and colleagues [97] reported elevated vitellogenin mRNA levels in both resident and caged brown trout downstream, but not upstream, of a wastewater treatment plant in a small Swiss stream. In contrast to the matching vitellogenin mRNA levels in these fish, vitellogenin protein expression was only upregulated in resident fish, but not caged males. The authors hypothesized that this may be related to the differing exposure history of the two study populations [97]. Second, caged fish were in excellent physiological condition at the onset of the study. They were all hatchery reared with *ad libitum* feed until caging, and had near optimum growing conditions at the hatchery. The better nutritional status of caged sunfish may contribute to the greater plasma vtg concentrations observed in caged fish in comparison to male resident sunfish from the same sites (Table 4). A better nutritional status may allow for greater resource allocation to protein biosynthesis, especially in caged fish that were caged in the effluent plumes of wastewater treatment plant discharges.

When significant biological effects were observed between reaches or sample sites, their frequency was similar for female and male fish, whether in resident or caged fish. Among the seven endpoints that were statistically analyzed (pathologies were sporadically found in all treatments with no strong patterns), two of the three morphologic indices and staples of fisheries biology (CF, GSI) were the least frequently affected. In caged fish, in particular, these two indices provided little evidence of biological effects even when fish livers documented clear evidence of a chemical insult (i.e., changes in relative liver size and prominence of hepatocyte vacuoles), as was evident in the RDA. The muted response of CF and GSI may either reflect adaptive changes in resident fish [98] or the short exposure duration of the caged fish that had been well fed until deployment and may have had sufficient reserves to buffer the exposure stress. Livers, in contrast, serve as the principal detoxifying organ and respond rapidly to a chemical insult, often resulting in more prominent vacuoles in liver hepatocytes [99, 100]. Because of the sensitivity and rapidness of change, liver vacuolization has been recommended as a measure of contaminant exposure [101].

### Biological consequences of CEC exposure

RDA identified the response variable of plasma glucose concentrations, an indicator of metabolic stress [39], as a sensitive biomarker of CEC exposure in both resident and caged sunfish. Changes to the bioenergetics status as a result of contaminant exposure have been noted in previous studies including bluegill sunfish [39, 66]. While Bevelhimer and colleagues [39] noted differences in glucose concentrations between wild-caught fish captured in areas affected by a coal ash spill, Adams and colleagues [66] noted changes in triglyceride concentrations in sunfish across a contaminant gradient. Both measures, glucose and triglyceride, are considered bioindicators of energetic stress and changes to metabolic processing. In the current study, the use of glucose as indicator was chosen due to its previous documented utility [39] and due to the ease of use and reliability of glucose monitors given the large number of processed fish samples. Given the prominence of alterations in blood glucose concentrations in the current study, it is important to note that a previous study documented changes in glucose concentrations for up to two hours after fish were exposed to electroshocking stimuli but not regular handling [102]. In the current study, resident and caged sunfish were processed within eight hours of either electroshocking and handling (resident fish) or handling alone (caged fish). Since positive correlations between glucose concentrations, HSI and hepatocyte vacuoles, and negative correlations with CF and GSI were consistent across resident and caged sunfish, we may assume that the effect of electroshocking resident fish did not bias our results.

Despite the complexity of observed biological effects, the Fox River provided evidence of multi-endpoint effects that are biologically consistent with CEC exposure. In the Fox River, which had a large number of chemical classes detected in water and had the second highest EEQ value in sediment, increased relative liver size (HSI) was consistently observed in resident and caged fish independent of sex. This was paralleled by an increase in the prominence of hepatocyte vacuoles (and a decrease in CF for resident fish) from upstream to downstream. In the 2014 fish samples collected from the Raquette River, prominence of hepatocyte vacuoles decreased in the middle reach of the river in resident and caged fish, independent of sex, and was matched by a similar pattern for plasma vitellogenin and glucose concentrations. The concentrations of both of these molecules in blood are intrinsically connected to the liver as vitellogenin is synthesized by the liver in response to increased estrogen concentrations, while glucose is stored (as glycogen) in this organ and released as a result of metabolic stress.

Even with the above described examples of biologically consistent effects across endpoints, the overall association of CEC presence and biological effects was too multi-faceted to be fully comprehended by an analysis of variance of individual effects between sites within a river. Consequently, we used the RDA to identify patterns and associations among our data matrices and to reduce the overall complexity of the data set by eliminating co-variance. The value of this statistical tool becomes apparent when comparing the results between subsets of the overall data set (female vs. male; resident vs. caged fish). Plasma glucose concentrations consistently dominate the first axis (i.e., the axis with the greatest explanatory power) of the RDA, independent of fish origin (resident, caged) or sex, and is mostly consistent between matrices (water only; sediment only; combined water and sediment). Increased plasma glucose concentrations as a result of pollutant stress have been noted in previous studies of pesticide exposure [103, 104], estrogenic compound exposure [105] and whole effluent exposure [106]. The consistent opposition of plasma glucose concentration and relative gonad size (GSI) and gonad maturity along the first axis of the RDA suggests that increasing plasma glucose concentrations are inversely correlated with the reproductive potential of fish (Fig 6; S10 and S11 Figs). Reduction in the relative size of gonads is commonly found in fish from polluted riverine sites [107–109].

The linkage between energy balance (represented by plasma glucose concentrations in the current study) and reproduction is well established [110] (reviewed in [111]), as the finite amount of energy available to an organism has to be balanced between basic physiological needs and reproductive output. Heightened metabolic stress as a result of pollutant exposure increases core energetic needs at the expense of reproductive output [110, 112]. The energetic costs of CEC exposure are further demonstrated by the opposition of CF and HSI/hepatocyte vacuolization on the second axis of the redundancy analysis. Here again, increased energetic expenses associated with stress response are inversely associated with a reduction of CF and the overall nutritional health of the organism.

In contrast to the prominence of the above mentioned biological endpoints, plasma vitellogenin concentrations and tissue pathologies played only a minor and more variable role in the variability of the data matrix. Patterns for these endpoints varied between resident and caged fish and between water and sediment matrices. Given the focus on plasma vitellogenin concentrations in many CEC studies, especially estrogenic CECs, we expected a greater prominence for this endpoint in our analysis, particularly in male fish. However, total estrogenicity (EEQ) seldom reached concentrations beyond 20 ng/L. Only three of the 27 sites had measured EEQs greater than 20 ng/L, and only seven sites were above 10 ng/L (Table 2). Therefore, plasma vitellogenin synthesis would unlikely be induced in male fish based on previous laboratory studies [89, 113].

Pathologies were too evenly spread across fish and sites to provide much insight. In the RDA, pathologies only took on a more prominent role when associated with sediment chemistry, perhaps reflecting the effects of hydrophobic compounds bound to sediment. It is, however, noteworthy that in 72% of resident fish at least one pathology (most frequently parasites in the liver) was observed. The effect of existing interactions between CEC exposures and pathologies, particularly parasite loads, represents a critical knowledge gap–especially considering that the liver is the main detoxifying organ of the organism and parasitic infestations are common in fish exposed to estrogenic CECs [114].

RDA identified several significant explanatory variables including pharmaceuticals, fragrances, and PAHs. Chemicals in all three classes have been implicated in previous studies as causing endocrine disruption in exposed fishes [58, 115, 116, 117]. While pharmaceuticals were identified as a significant explanatory variable when all chemicals were evaluated, a secondary RDA evaluating only the contribution of pharmaceutical (grouped into subclasses with similar mode-of-action) did not identify any of the subclasses as having strong explanatory power. This suggests that the totality of pharmaceutical exposures adversely affected fish. Lastly, it is noteworthy that other abiotic and biotic factors that were not enumerated in the current study may have contributed to the observed effects. Higher pollutant loads are frequently associated with other physico-chemical and physical alterations in the environment (i.e., greater nitrification of waters, reduced dissolved oxygen, greater turbidity, declining habitat quality and availability). Beyond the scope of the current study, the interactions of multiple stressors, including CECs, is urgently needed to comprehensively assess the impact of CECs on the sustainability of fish populations [2, 90]. Particularly in the Great Lakes Basin, aquatic organisms are exposed to dynamic variations in abiotic factors throughout the year, such as temperature and salinity, in combination with the variability in pollutant loads, which may be related to observed effects.

### Experimental design considerations

The current study examined over 2250 sunfish across 27 sites. Despite this effort, it proved difficult to consistently obtain the desired double-digit sample sizes for robust statistical analysis of female and male resident and caged fish [38]. The variability and complexity of aquatic ecosystems remains a challenge for any field study. Although an attempt was made to conduct all field studies in a short time frame to match environmental conditions (for example water temperature) that influence the expression of biological effects in exposed organisms, physico-chemical differences between rivers and stream reaches are unavoidable and will add complexity to the data matrix. For example, urban watersheds usually have higher average temperatures than rural watersheds and stream reaches below major wastewater treatment plant outfalls contain greater nutrient loads than putative reference sites in forested upstream habitats. Consequently, the RDA analyses, while consistent in identifying prominent biological response variables, only explained approximately half of the variation in biological responses. Adding to this natural complexity, the individual variability [118] found in every population further complicates meaningful analysis of the resultant data matrices. This variability requires the use of statistical techniques capable of integrating these data sets. It also requires the investigator to use best professional judgment to reduce the complexity of the matrices whenever possible (for example, by combining chemicals into “CEC classes” and by collapsing the genus *Lepomis* into a single entity) while recognizing that, especially for CECs, these judgement calls remain tenuous. In the current study, RDA provided an integration of two complex matrices that resulted in the identification of patterns that would have easily been lost using less integrative methods (for example general linear models). However, multivariate techniques are limited by their need for large data matrices to draw from. These limitations highlight the need to develop comprehensive experimental designs to improve our understanding of the effects of CECs in aquatic ecosystems. Mesocosm studies, on-site exposures using temporary ad-hoc laboratory spaces, or non-lethal sampling techniques may be required to close some of the existing knowledge gaps.

The current study illustrates a common pattern of CEC presence in tributaries to the Laurentian Great Lakes that is most pronounced in urban influenced aquatic ecosystems but that is also sufficiently widespread to achieve near omnipresence in the Great Lakes watershed. Biological effects were highly variable and frequently failed to follow an upstream-downstream pattern, but carried a consistent energetic cost as suggested by the strong plasma glucose response that may impact the reproductive potential of exposed fish [110] (reviewed in [111]). Although a field-based study of chemical presence and biological effects cannot attribute causality, the omnipresence of CECs and the documented biological effects support further study to predict causality with confidence.

The Laurentian Great Lakes have been a focal point for understanding legacy contaminants and subsequent remediation for decades [1]. More recently, there has been an increased focus on lakes due, in part, to algal blooms from increased nutrient runoff, increases in invasive species, and increased warming of water temperatures. The realization that the same ecosystem is also impacted by the presence of CECs is disturbing, but not unexpected. The Great Lakes Basin is home to diverse fish and wildlife habitats, and supports an economically valuable recreational and commercial fishery. Resource managers need to be aware of the multiple stressors that may be impacting their resources. Our study raises awareness of the risks that CEC may be posing to these resources, especially in areas with greater human influence and urbanization. With the consistently changing environment, and the abundance of stressors that may be present, CECs represent another threat that needs to be managed to protect and conserve the fish and wildlife resources in the Laurentian Great Lakes.

## Conclusions

This study correlated an extensive matrix of CECs in six Great Lakes tributaries with an equally extensive matrix of biological responses in resident and caged sunfish. The study confirmed an almost ubiquitous presence of CECs that calls into question the presence of relevant reference sites in the Great Lakes watershed. The complexity of the chemical occurrence data was matched by the complexity of the biological responses, which exhibited few consistent patterns of change when assessed solely between reaches or sites within a river or between rivers. However, full integration of both explanatory chemical variables and biological response variables revealed consistent biological effects of CEC exposure. Chief among them was a change in blood glucose concentration, an indicator of bioenergetics and stress, which was associated with increased relative liver size and greater prominence of hepatocyte vacuoles. These indicators of pollutant exposure were inversely correlated with indicators of reproductive potential, including smaller gonad size and less mature gametes. The current study highlights the need for greater integration of chemical and biological studies and suggests that CECs in the Laurentian Great Lakes Basin may adversely affect the reproductive potential and sustainability of exposed fish populations.

## Supporting information

S1 Fig Liver histology. Representative micrographs of liver histology representing four grades of liver vacuole prominence. (A) grade 1 –few visible liver vacuoles; (B) grade 2 –liver vacuoles visible but infrequent; (C) grade 3 –liver vacuoles widespread; (D) grade 4 –severe vacuolization. Liver vacuoles to the right of arrow heads; scale bar = 50μm distance.
(TIF)

S2 Fig Gonad histology. Representative gonad histology for (A) male and (B) female gonad tissue. **pn** perinuclear oocyte; **ca** cortical alveolar oocyte; **ev** early vitellogenic oocyte; **lv** late vitellogenic oocyte; **sg** spermatagonia; **sc** spermatocyte; **st** spermatid; **sz** spermatozoa (mature sperm); Scale bar = 50μm.
(TIF)

S3 Fig Pathologies. Histological pathologies counted as presence/ absence for statistical analysis. (A) liver with eosinophilic fluids (right of arrow head); (B) liver with parasite; (C) ovary with eosinophilic fluids; (D) ovary with parasite; (E) testis with eosinophilic fluids; (F) testis with parasite. Scale bar = 50μm.
(TIF)

S4 Fig Stacked water chemistry. Total chemical concentrations (μg/L) in water samples collected from sites in the Great Lakes Basin. Values above stacked bars represent the number of chemicals detected at that site in water samples. Chemicals representing each class are presented in S2 Table.
(TIF)

S5 Fig Stacked sediment chemistry. Total chemical concentrations (μg/kg) in sediment samples collected from sites in the Great Lakes Basin. Values above stacked bars represent the number of chemicals detected at that site in sediment samples. Chemicals representing each class are presented in S2 Table.
(TIF)

S6 Fig Biological endpoints in resident female sunfish. (A) condition factor; (B) gonadosomatic index; (C) gonad maturity ranked on a scale of 0 to 4; (D) sum of pathologic observations on a scale of 0 to 7; (E) hepatosomatic index. Sample river location located above panels (A) and (B), with columns representing upstream to downstream within each river from left to right. Specific sample site identification can be found in Table 1. Column graphs indicate mean + standard deviation in panels (A), (B), and (D). Box-and-whisker plots indicate range, 25^th^ and 75^th^ percentiles, and mean values in panel (C). Statistical significance (Kruskal-Wallis with Dunn’s post-test; p<0.05) within panels are identified by letters, with the p-value identified below graphs. Sample size provided in S3 Table.
(TIF)

S7 Fig Biological endpoints in resident male sunfish. (A) condition factor; (B) gonadosomatic index; (C) gonad maturity ranked on a scale of 0 to 4; (D) sum of pathologic observations on a scale of 0 to 7. Sample river location located above panels (A) and (B), with columns representing upstream to downstream within each river from left to right. Specific sample site identification can be found in Table 1. Column graphs indicate mean + standard deviation in panels (A), (B), and (D). Box-and-whisker plots indicate range, 25^th^ and 75^th^ percentiles, and mean values in panel (C). Statistical significance (Kruskal-Wallis with Dunn’s post-test; p<0.05) within panels are identified by letters, with the p-value identified below graphs. Sample size provided in S3 Table.
(TIF)

S8 Fig Biological endpoints in caged female sunfish. (A) condition factor; (B) gonadosomatic index; (C) gonad maturity ranked on a scale of 0 to 4; (D) sum of pathologic observations on a scale of 0 to 7. Sample river location located above panels (A) and (B), with columns representing upstream to downstream within each river from left to right. Specific sample site identification can be found in Table 1. Column graphs indicate mean + standard deviation in panels (A), (B), and (D). Box-and-whisker plots indicate range, 25^th^ and 75^th^ percentiles, and mean values in panel (C). Statistical significance (Kruskal-Wallis with Dunn’s post-test; p<0.05) within panels are identified by letters, with the p-value identified below graphs. Sample size provided in S3 Table.
(TIF)

S9 Fig Biological endpoints in caged male sunfish. (A) condition factor; (B) gonadosomatic index; (C) gonad maturity ranked on a scale of 0 to 4; (D) sum of pathologic observations on a scale of 0 to 7. Sample river location located above panels (A) and (B), with columns representing upstream to downstream within each river from left to right. Specific sample site identification can be found in Table 1. Column graphs indicate mean + standard deviation in panels (A), (B), and (D). Box-and-whisker plots indicate range, 25^th^ and 75^th^ percentiles, and mean values in panel (C). Statistical significance (Kruskal-Wallis with Dunn’s post-test; p<0.05) within panels are identified by letters, with the p-value identified below graphs. Sample size provided in S3 Table.
(TIF)

S10 Fig Canonical redundancy analysis for sediment samples and biological results. (A) Resident females, (B) Resident males, (C) Caged females, and (D) Caged males. Number in parentheses on axes indicate the percent variability that is explained by that axis. Sample site information can be found in Table 1. Sample class information can be found in Table 3. Biological response information can be found in Table 4.
(TIF)

S11 Fig Canonical redundancy analysis results for both matrices (water and sediment) together (8) and biological results. (A) Resident females, (B) Resident males, (C) Caged females, and (D) Caged males. Colors for numbers indicate chemical source (Red = Water, Green = Sediment), and numbers indicate corresponding chemical class (1 = Sum EEQ, 2 = Sterol, 3 = Plasticizer, 4 = Phenolic, 5 = Pharmaceutical, 6 = Pesticide, 7 = Polycyclic Aromatic Hydrocarbon, 8 = Other, 9 = Organohalide, 10 = Insect Repellent, 11 = Industrial, 12 = Hormone, 13 = Fragrance, 14 = Flame Retardant, 15 = Fecal Indicator, 16 = Alkylphenol). Number in parentheses on axes indicate the percent variability that is explained by that axis. Sample site information can be found in Table 1. Sample class information can be found in Table 2a and 2b. Biological response information can be found in Table 4.
(TIF)

S12 Fig Canonical redundancy analysis results for pharmaceutical subclasses in water samples and biological results. (A) Resident females, (B) Resident males, (C) Caged females, and (D) Caged males. Number in parentheses on axes indicate the percent variability that is explained by that axis. Sample site information can be found in Table 1. Sample subclass information can be found in S2 Table. Biological response information can be found in Table 4.
(TIF)

S1 Table Sampling site information. Additional sample site information, including sample site ID, river reach section, latitude, longitude, sample site station name, identification of nearest city or municipality to the sample site, 2014 population and population density (person/km^2^) in the drainage basin, and dates of field sample events for each station.
(XLSX)

S2 Table Composition of chemical classes used in RDA analysis. (A) List of chemicals representing each chemical class, pharmaceutical subclass, and estradiol equivalency multiplier used to determine estrogenic equivalency value.
(XLSX)

S3 Table Sample size for fish analyzed in the current study. Sample size may vary slightly between biological endpoints due to factors such as insufficient tissues samples or other post-dissection effects.
(XLSX)

S4 Table P-values for biological data. P-values for biological endpoints with statistical significance between sites. Biological response information can be found in Figs 2–5 and Table 4.
(XLSX)

S5 Table RDA analysis. Constrained and unconstrained values from the canonical redundancy analysis for resident and caged male and female sunfish from 2013 and 2014 samples.
(XLSX)

S1 File R Statistical code. R code developed in the Vegan software package for R.
(DOCX)

S2 File Raw biological response data of fish collected in 2013 and 2014. (*Microsoft Excel*) All biological data used in this study including: fish number, sample year, sample river, the sample river reach section, identification of caged or resident fish, genus, common species name, total length (mm), total weight (g), gonadal weight (g), liver weight (g), condition factor (CF), gonadal somatic index (GSI), hepatosomatic index (HSI), plasma vitellogenin concentration (μg/mL), log_10_ plasma vitellogenin concentration, plasma glucose concentration (mg/dL), sex of fish based on histological analysis, percent of spermatogonia or number of immature oocytes, percent of spermatocytes or number of perinuclear oocytes, percent of spermatids or number of early vitellogenic oocytes, percent of spermatozoa or number of mature oocytes, presence of eosinophilic fluid (1 = yes, 0 = no), gonad maturity (GPA) ranked on a scale of 0 to 4, prevalence of vacuoles in hepatocytes ranked on a severity scale of 1 to 4, presence of liver pathologic features (1 = yes, 0 = no), presence of gonadal abnormality (1 = yes, 0 = no), presence of parasites in the gonads (1 = yes, 0 = no), presence of parasites in the liver (1 = yes, 0 = no), presence of macroscopic pathologic features (1 = yes, 0 = no), sum of pathologic observations on a scale of 0 to 7, and presence of pathologic abnormalities (1 = yes, 0 = no).
(XLSX)
