# Supplementary figures and images for: Contaminants of emerging concern in tributaries to the Laurentian Great Lakes: II. Biological consequences of exposure
Source: PLoS One. 2017 Sep 27;12(9):e0184725. doi: 10.1371/journal.pone.0184725 (PMC5617166; doi:10.1371/journal.pone.0184725)

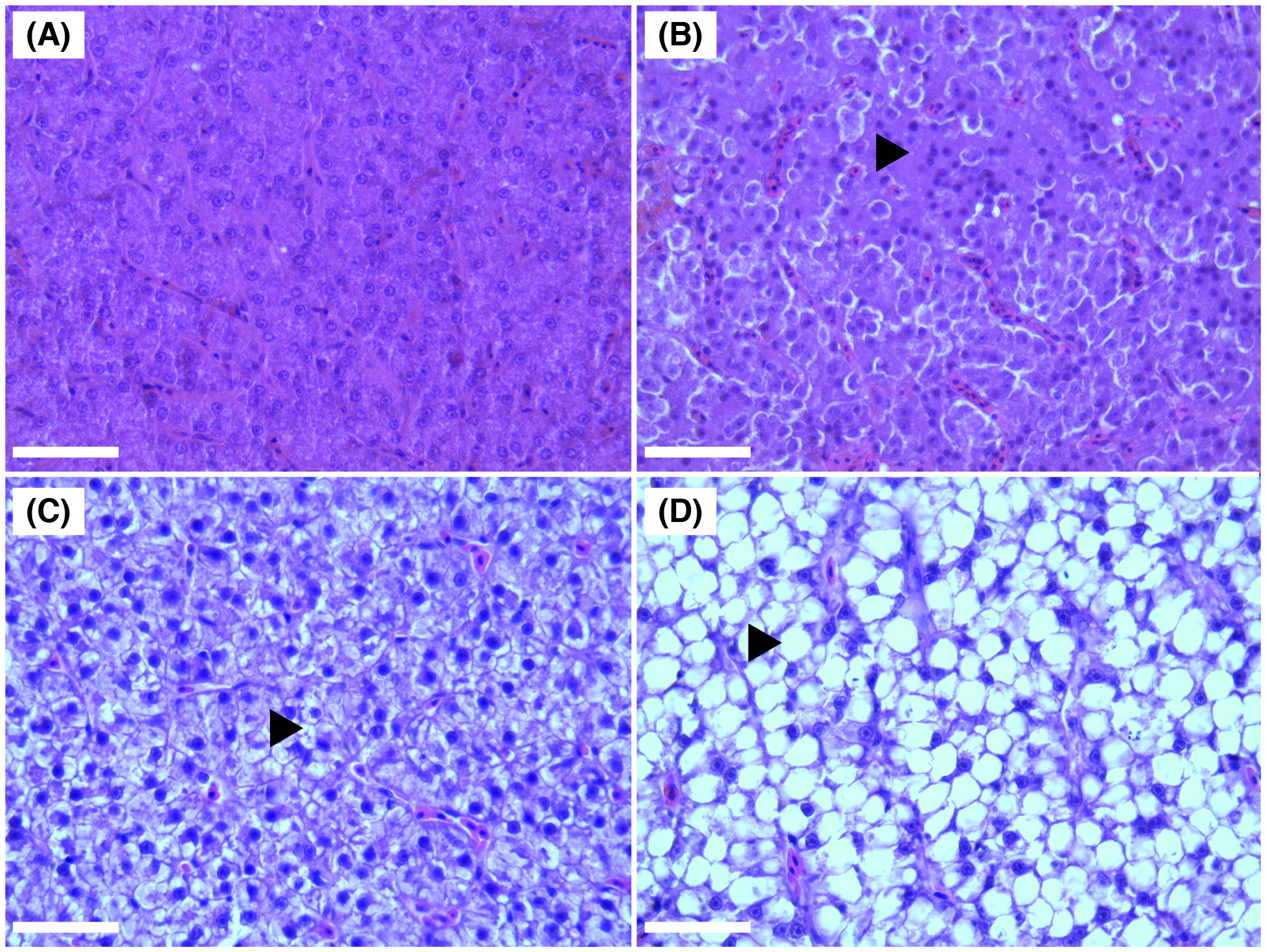

Supplement: S1 Fig — Representative micrographs of liver histology representing four grades of liver vacuole prominence. (A) grade 1 –few visible liver vacuoles; (B) grade 2 –liver vacuoles visible but infrequent; (C) grade 3 –liver vacuoles widespread; (D) grade 4 –severe vacuolization. Liver vacuoles to the right of arrow heads; scale bar = 50μm distance. (TIF) [file pone.0184725.s001.tif]

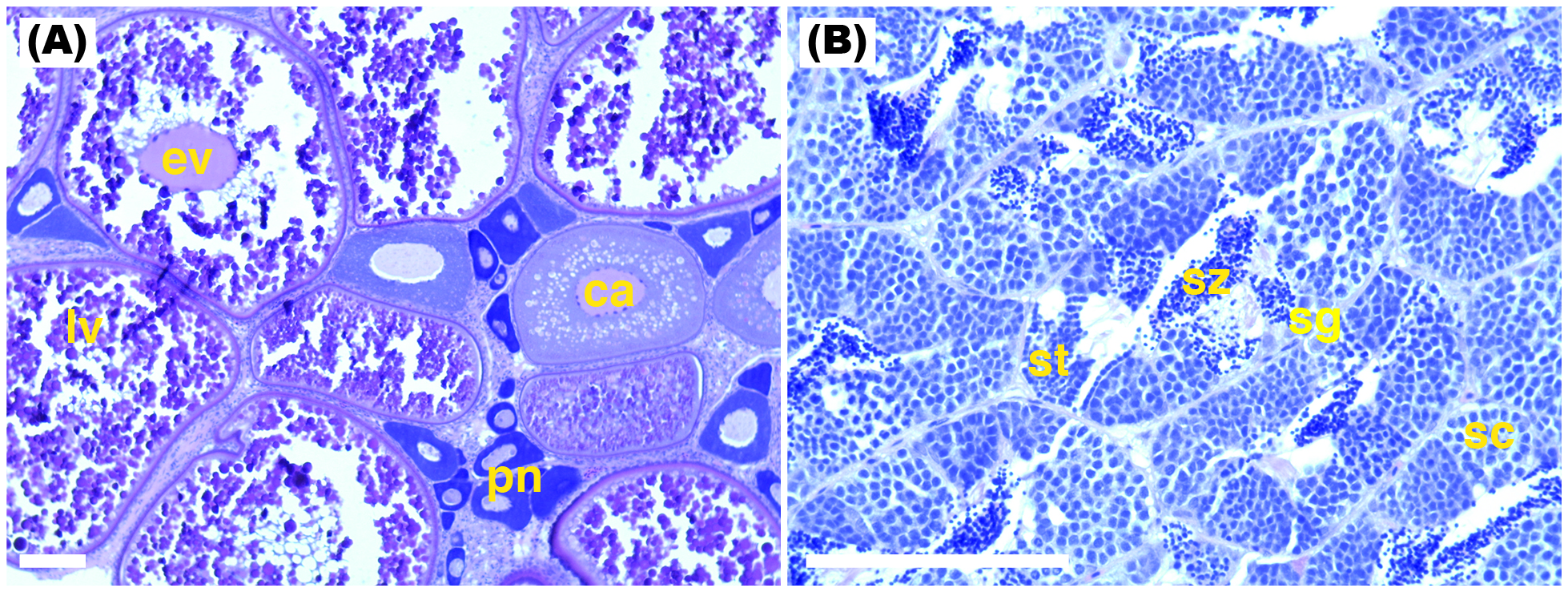

Supplement: S2 Fig — Representative gonad histology for (A) male and (B) female gonad tissue. pn perinuclear oocyte; ca cortical alveolar oocyte; ev early vitellogenic oocyte; lv late vitellogenic oocyte; sg spermatagonia; sc spermatocyte; st spermatid; sz spermatozoa (mature sperm); Scale bar = 50μm. (TIF) [file pone.0184725.s002.tif]

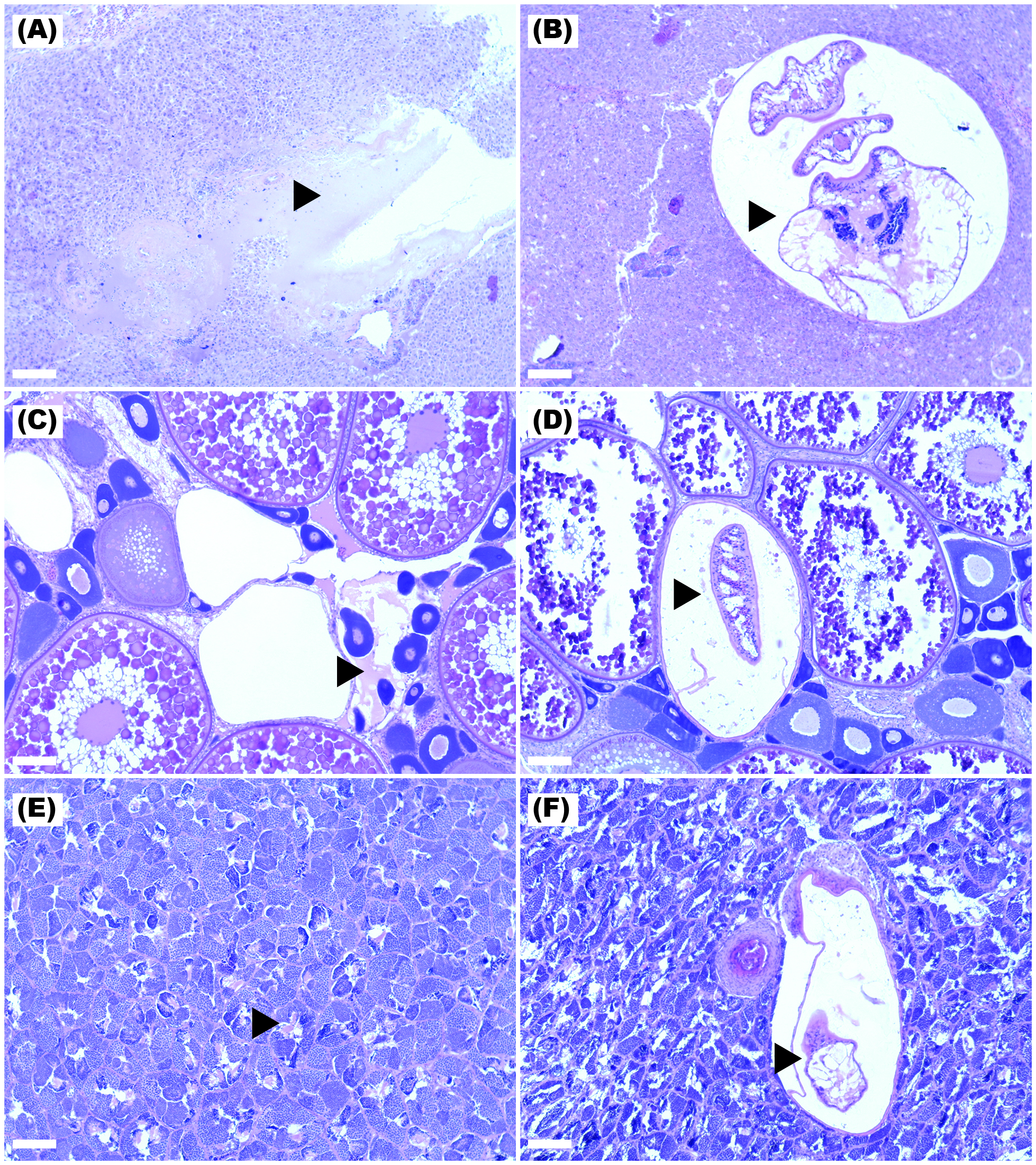

Supplement: S3 Fig — Histological pathologies counted as presence/ absence for statistical analysis. (A) liver with eosinophilic fluids (right of arrow head); (B) liver with parasite; (C) ovary with eosinophilic fluids; (D) ovary with parasite; (E) testis with eosinophilic fluids; (F) testis with parasite. Scale bar = 50μm. (TIF) [file pone.0184725.s003.tif]

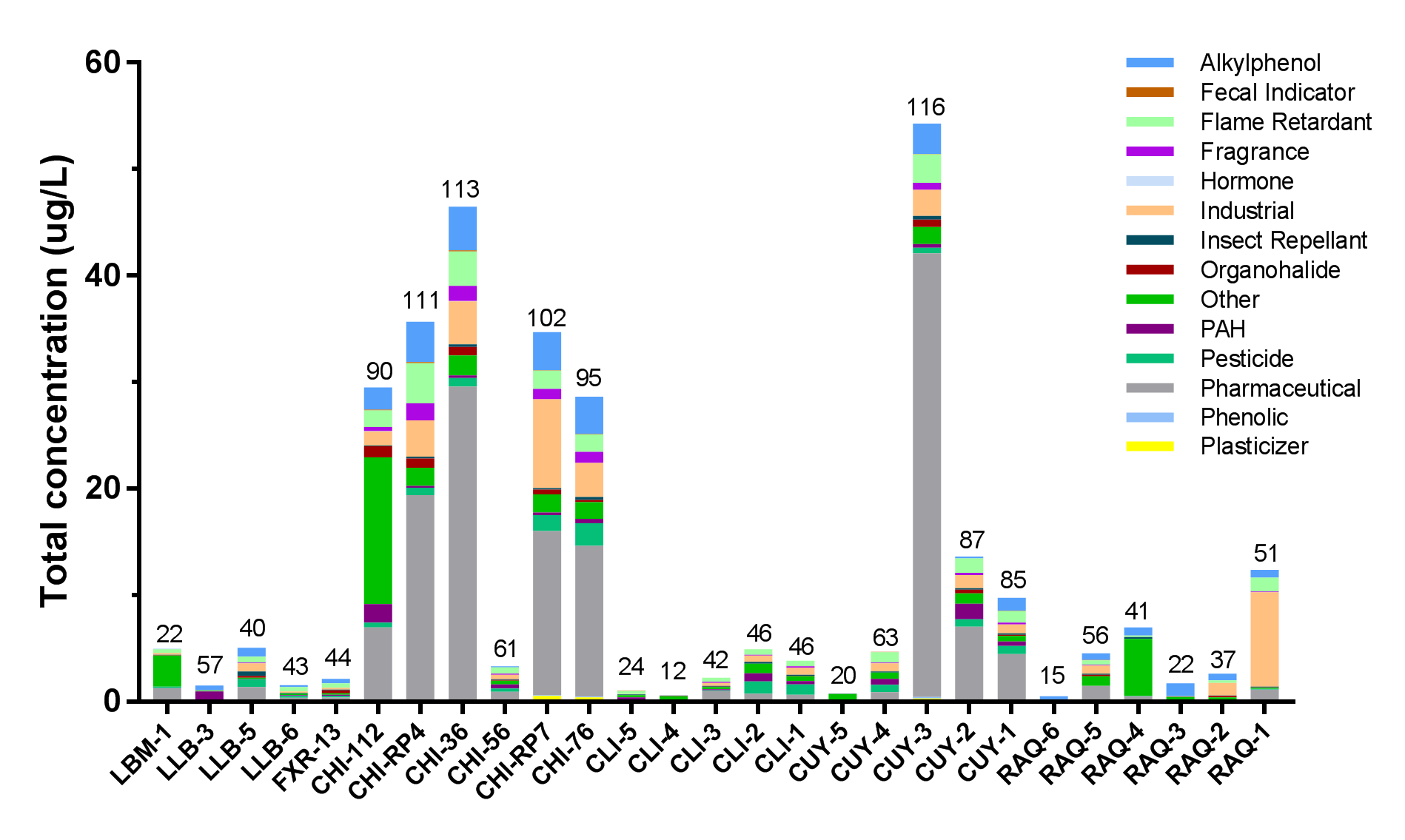

Supplement: S4 Fig — Total chemical concentrations (μg/L) in water samples collected from sites in the Great Lakes Basin. Values above stacked bars represent the number of chemicals detected at that site in water samples. Chemicals representing each class are presented in S2 Table. (TIF) [file pone.0184725.s004.tif]

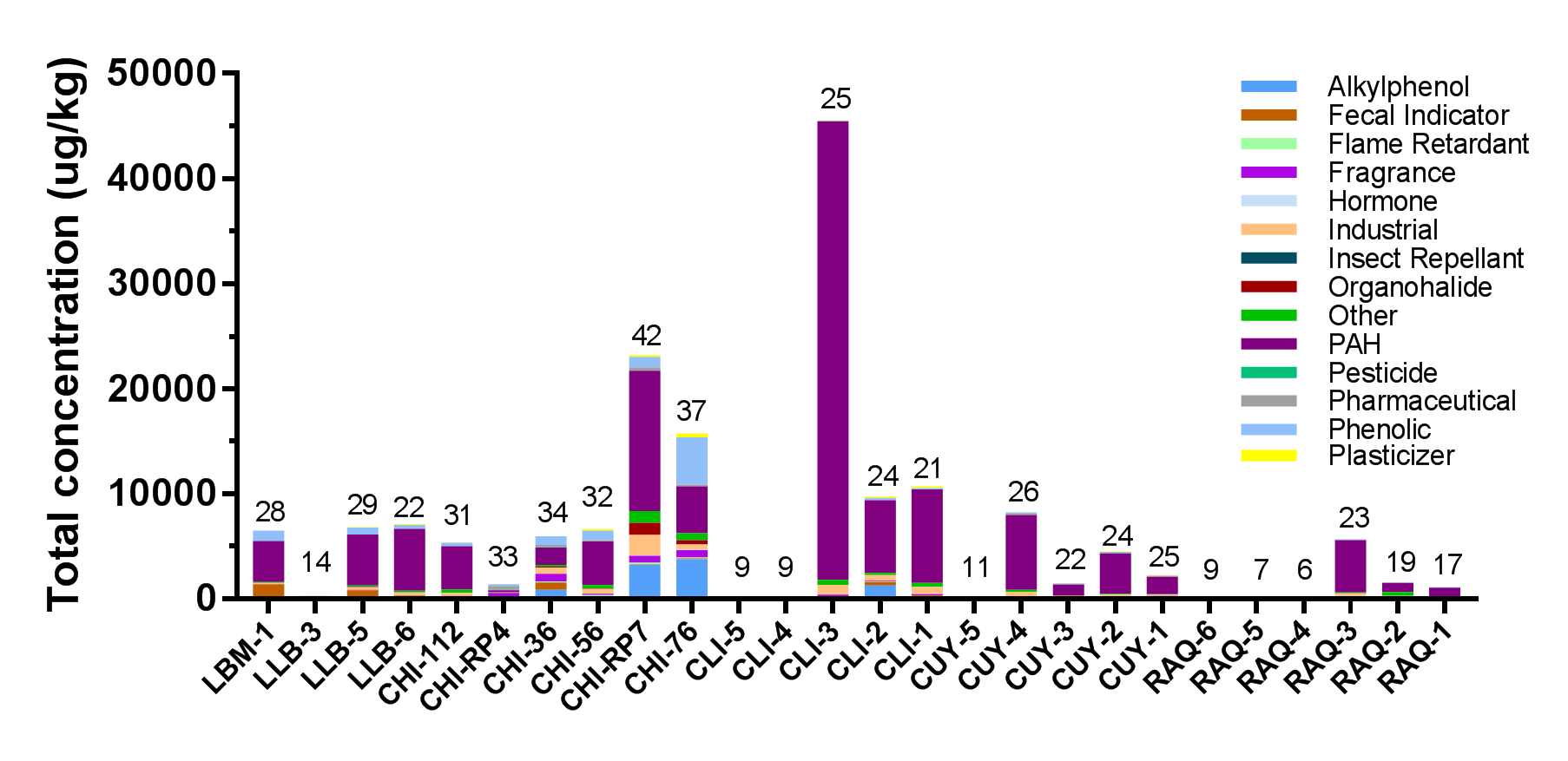

Supplement: S5 Fig — Total chemical concentrations (μg/kg) in sediment samples collected from sites in the Great Lakes Basin. Values above stacked bars represent the number of chemicals detected at that site in sediment samples. Chemicals representing each class are presented in S2 Table. (TIF) [file pone.0184725.s005.tif]

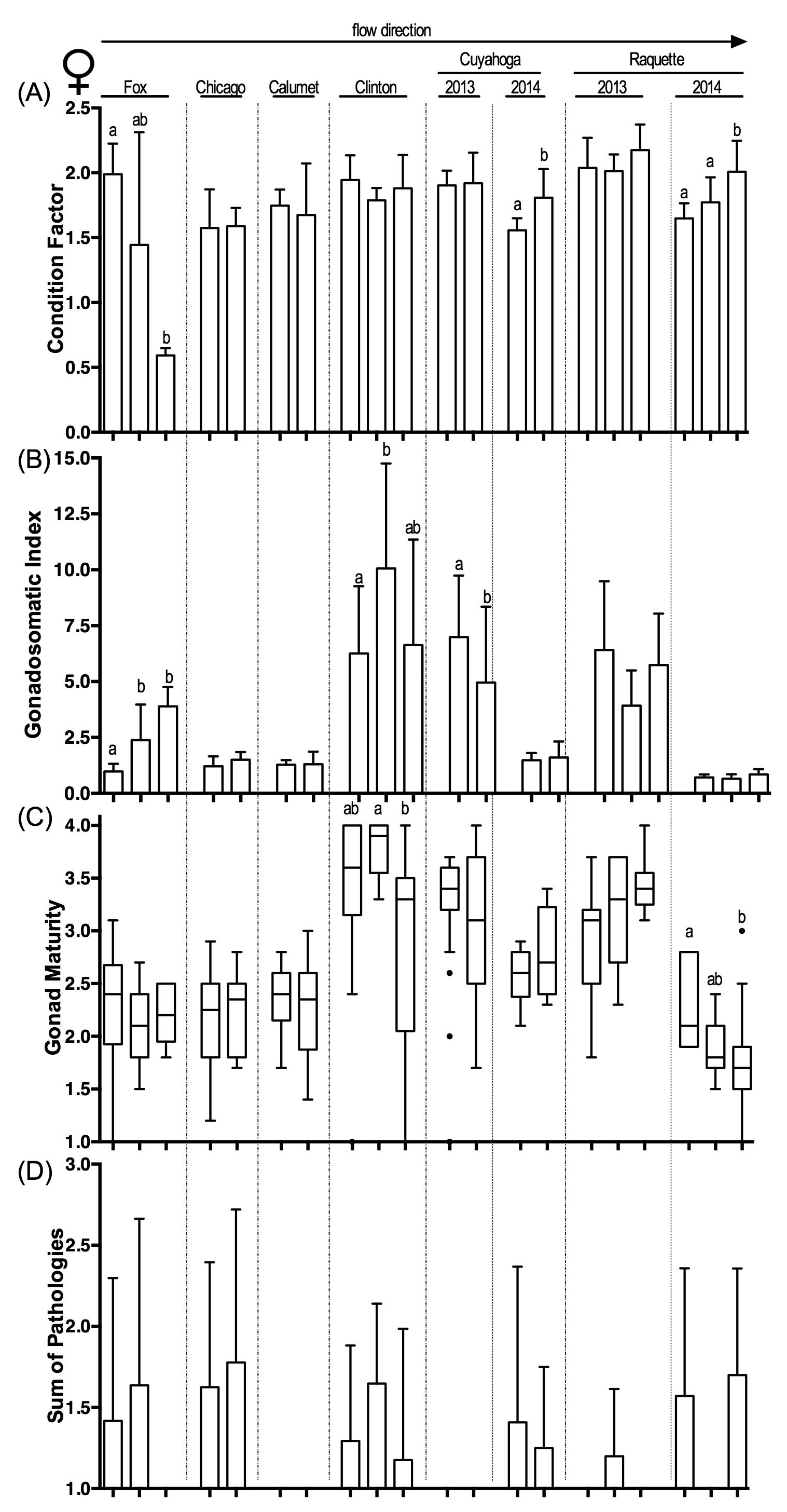

Supplement: S6 Fig — (A) condition factor; (B) gonadosomatic index; (C) gonad maturity ranked on a scale of 0 to 4; (D) sum of pathologic observations on a scale of 0 to 7; (E) hepatosomatic index. Sample river location located above panels (A) and (B), with columns representing upstream to downstream within each river from left to right. Specific sample site identification can be found in Table 1. Column graphs indicate mean + standard deviation in panels (A), (B), and (D). Box-and-whisker plots indicate range, 25th and 75th percentiles, and mean values in panel (C). Statistical significance (Kruskal-Wallis with Dunn’s post-test; p<0.05) within panels are identified by letters, with the p-value identified below graphs. Sample size provided in S3 Table. (TIF) [file pone.0184725.s006.tif]

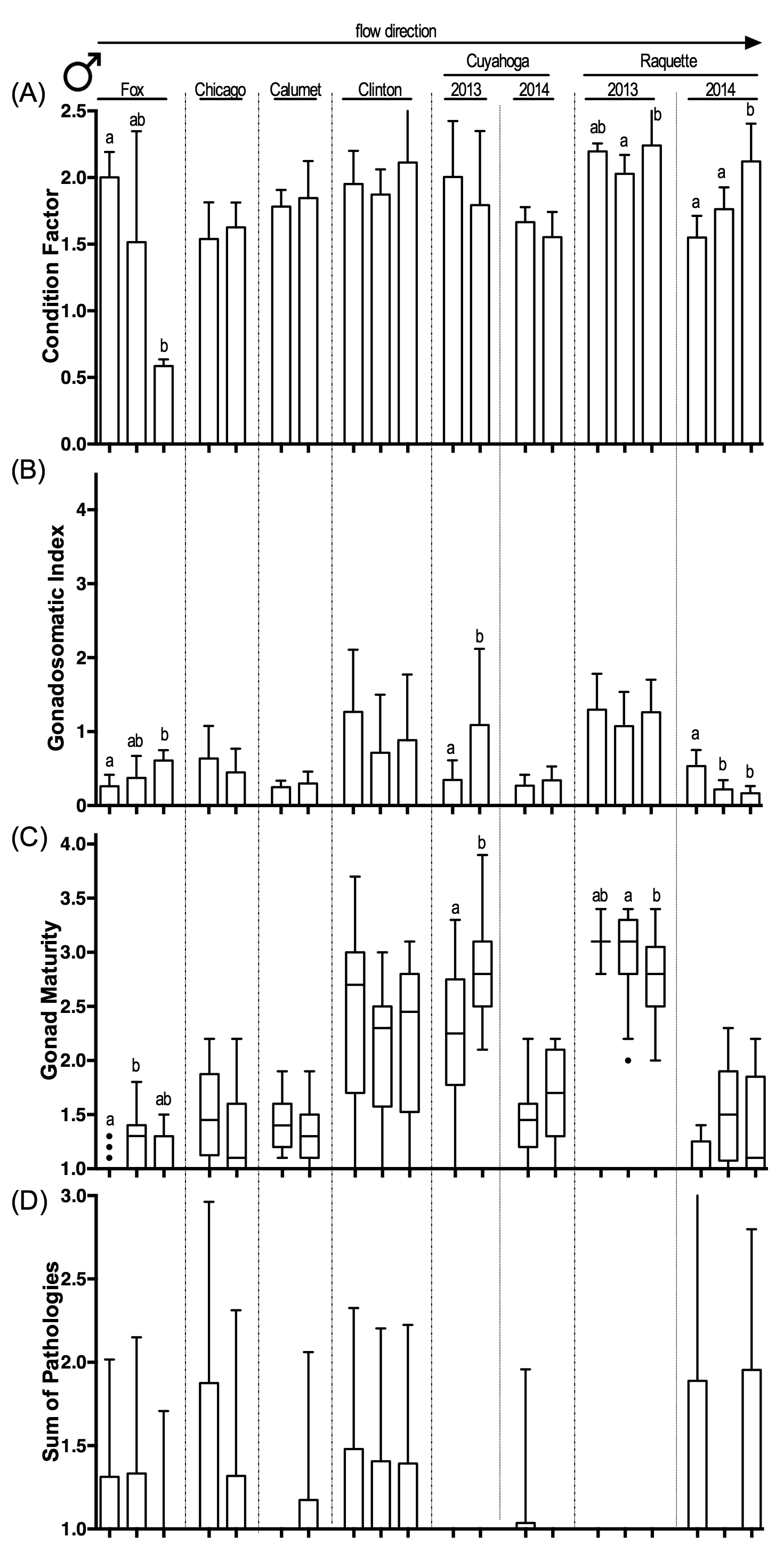

Supplement: S7 Fig — (A) condition factor; (B) gonadosomatic index; (C) gonad maturity ranked on a scale of 0 to 4; (D) sum of pathologic observations on a scale of 0 to 7. Sample river location located above panels (A) and (B), with columns representing upstream to downstream within each river from left to right. Specific sample site identification can be found in Table 1. Column graphs indicate mean + standard deviation in panels (A), (B), and (D). Box-and-whisker plots indicate range, 25th and 75th percentiles, and mean values in panel (C). Statistical significance (Kruskal-Wallis with Dunn’s post-test; p<0.05) within panels are identified by letters, with the p-value identified below graphs. Sample size provided in S3 Table. (TIF) [file pone.0184725.s007.tif]

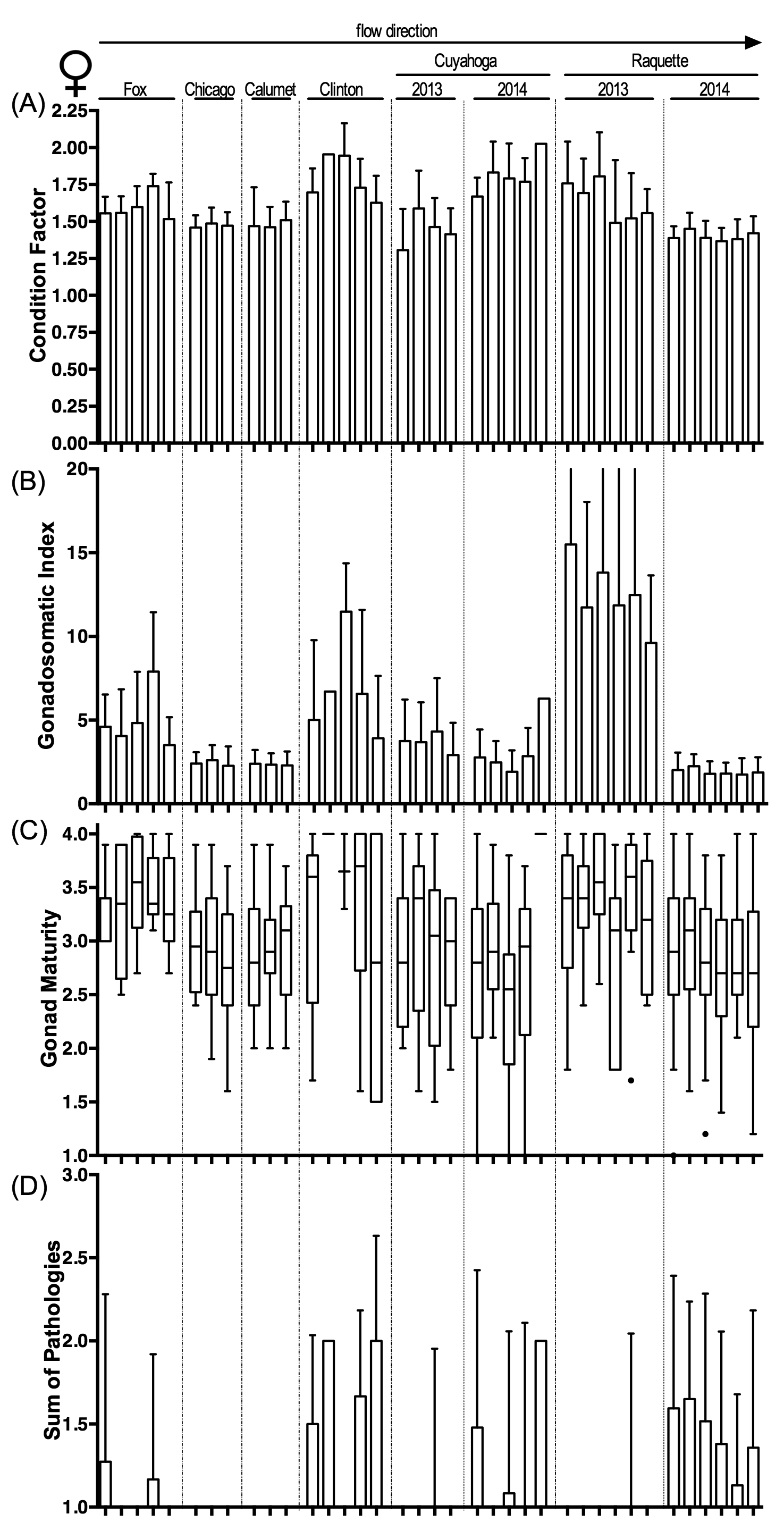

Supplement: S8 Fig — (A) condition factor; (B) gonadosomatic index; (C) gonad maturity ranked on a scale of 0 to 4; (D) sum of pathologic observations on a scale of 0 to 7. Sample river location located above panels (A) and (B), with columns representing upstream to downstream within each river from left to right. Specific sample site identification can be found in Table 1. Column graphs indicate mean + standard deviation in panels (A), (B), and (D). Box-and-whisker plots indicate range, 25th and 75th percentiles, and mean values in panel (C). Statistical significance (Kruskal-Wallis with Dunn’s post-test; p<0.05) within panels are identified by letters, with the p-value identified below graphs. Sample size provided in S3 Table. (TIF) [file pone.0184725.s008.tif]

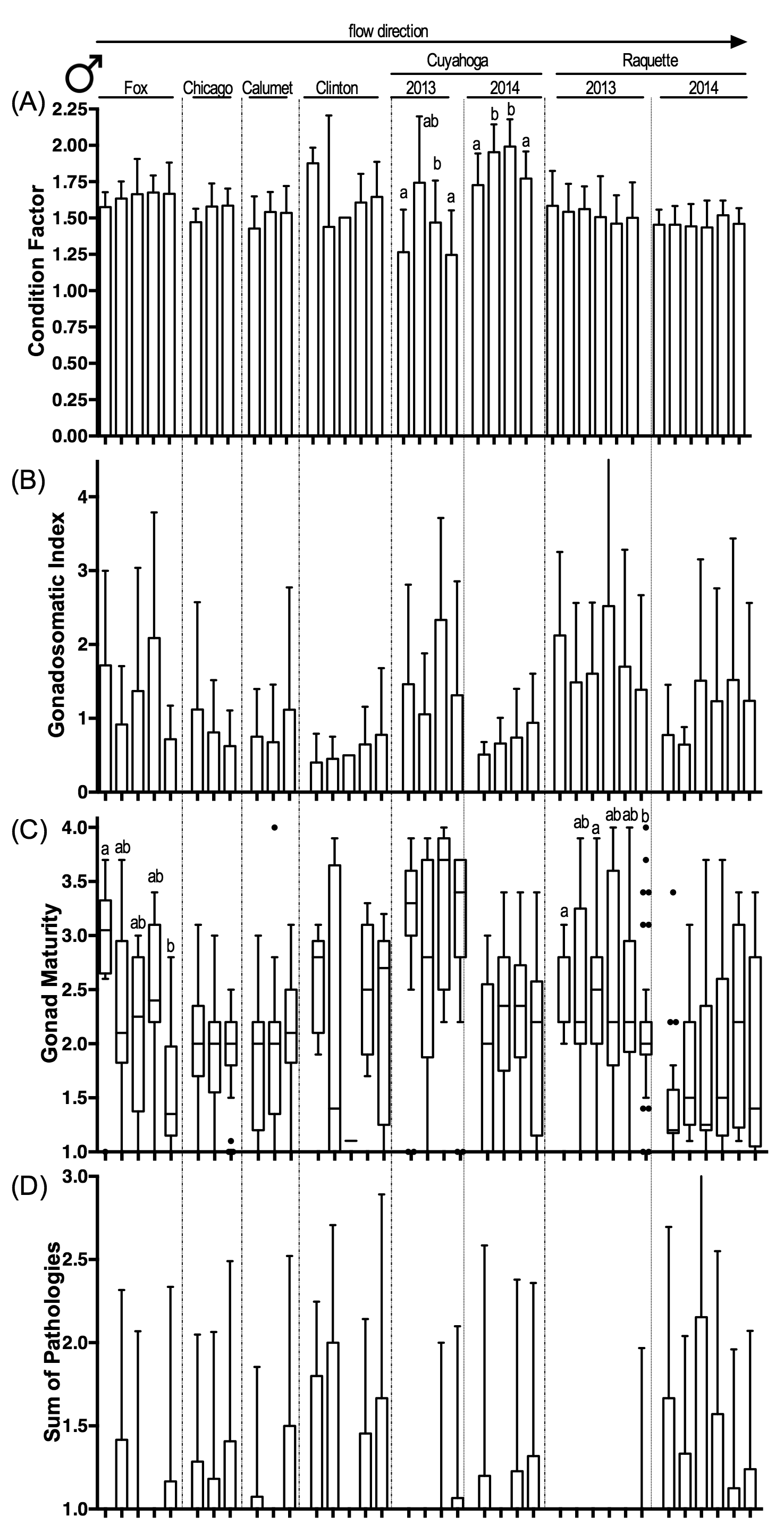

Supplement: S9 Fig — (A) condition factor; (B) gonadosomatic index; (C) gonad maturity ranked on a scale of 0 to 4; (D) sum of pathologic observations on a scale of 0 to 7. Sample river location located above panels (A) and (B), with columns representing upstream to downstream within each river from left to right. Specific sample site identification can be found in Table 1. Column graphs indicate mean + standard deviation in panels (A), (B), and (D). Box-and-whisker plots indicate range, 25th and 75th percentiles, and mean values in panel (C). Statistical significance (Kruskal-Wallis with Dunn’s post-test; p<0.05) within panels are identified by letters, with the p-value identified below graphs. Sample size provided in S3 Table. (TIF) [file pone.0184725.s009.tif]

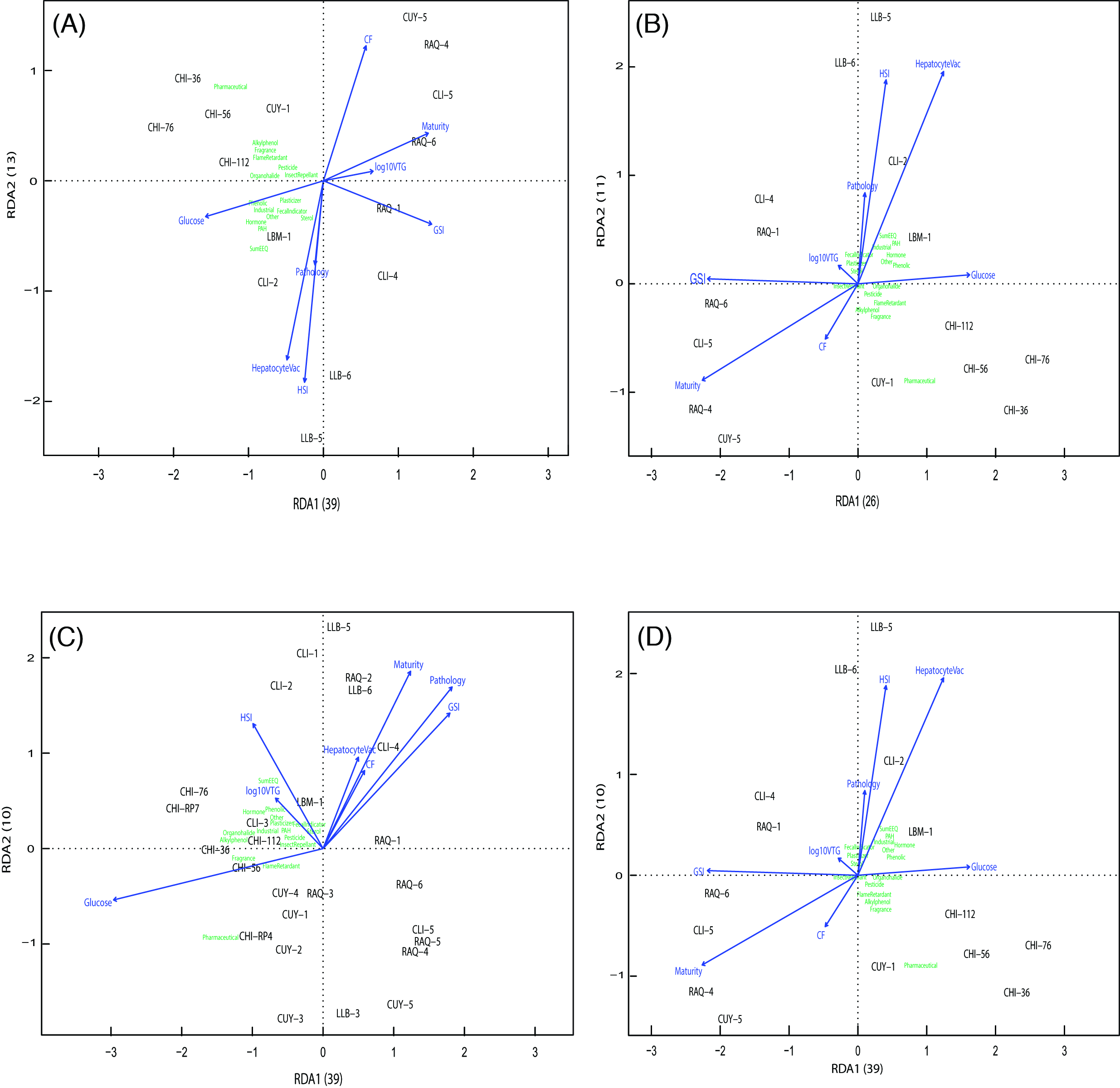

Supplement: S10 Fig — (A) Resident females, (B) Resident males, (C) Caged females, and (D) Caged males. Number in parentheses on axes indicate the percent variability that is explained by that axis. Sample site information can be found in Table 1. Sample class information can be found in Table 3. Biological response information can be found in Table 4. (TIF) [file pone.0184725.s010.tif]

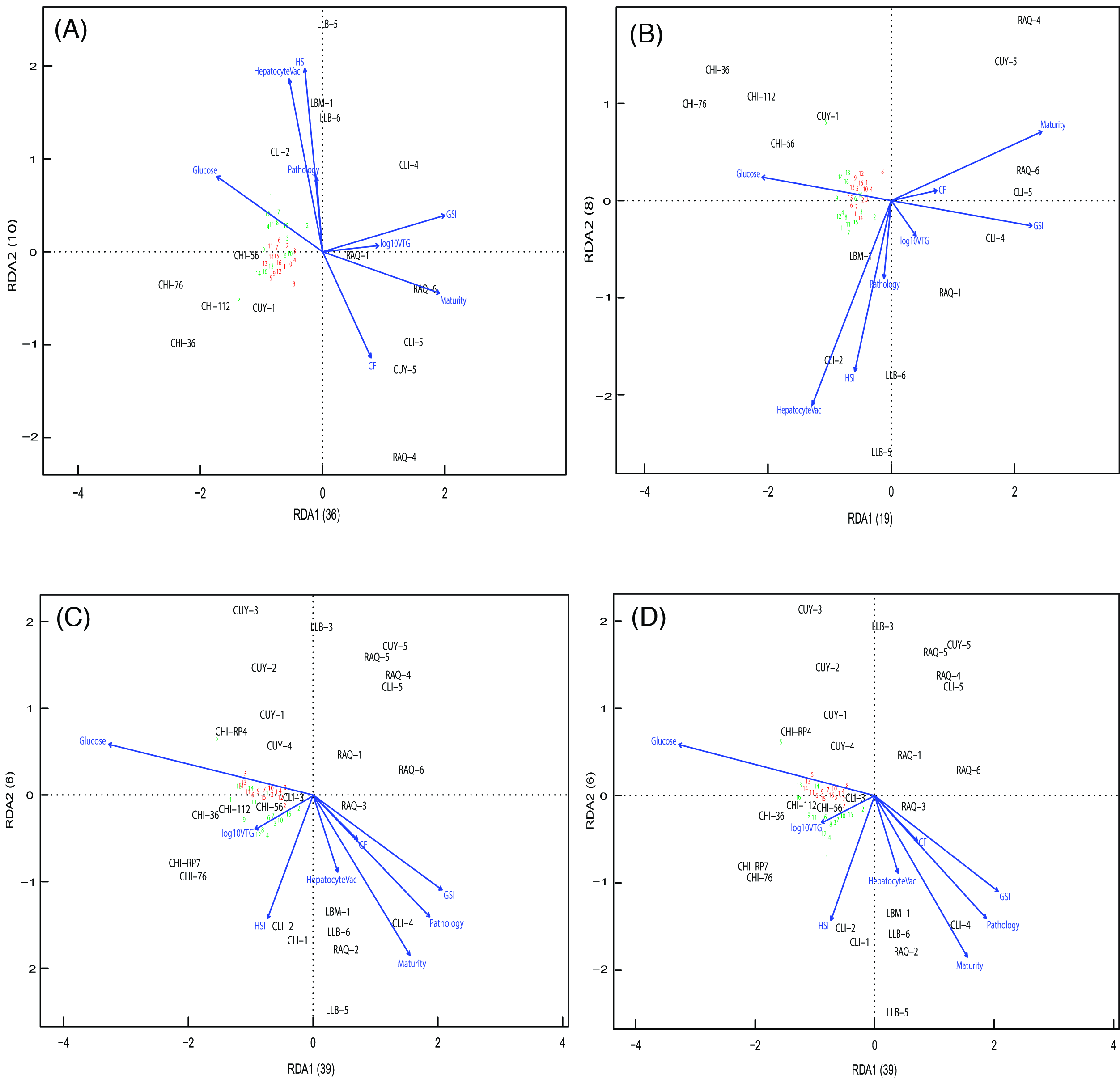

Supplement: S11 Fig — (A) Resident females, (B) Resident males, (C) Caged females, and (D) Caged males. Colors for numbers indicate chemical source (Red = Water, Green = Sediment), and numbers indicate corresponding chemical class (1 = Sum EEQ, 2 = Sterol, 3 = Plasticizer, 4 = Phenolic, 5 = Pharmaceutical, 6 = Pesticide, 7 = Polycyclic Aromatic Hydrocarbon, 8 = Other, 9 = Organohalide, 10 = Insect Repellent, 11 = Industrial, 12 = Hormone, 13 = Fragrance, 14 = Flame Retardant, 15 = Fecal Indicator, 16 = Alkylphenol). Number in parentheses on axes indicate the percent variability that is explained by that axis. Sample site information can be found in Table 1. Sample class information can be found in Table 2a and 2b. Biological response information can be found in Table 4. (TIF) [file pone.0184725.s011.tif]

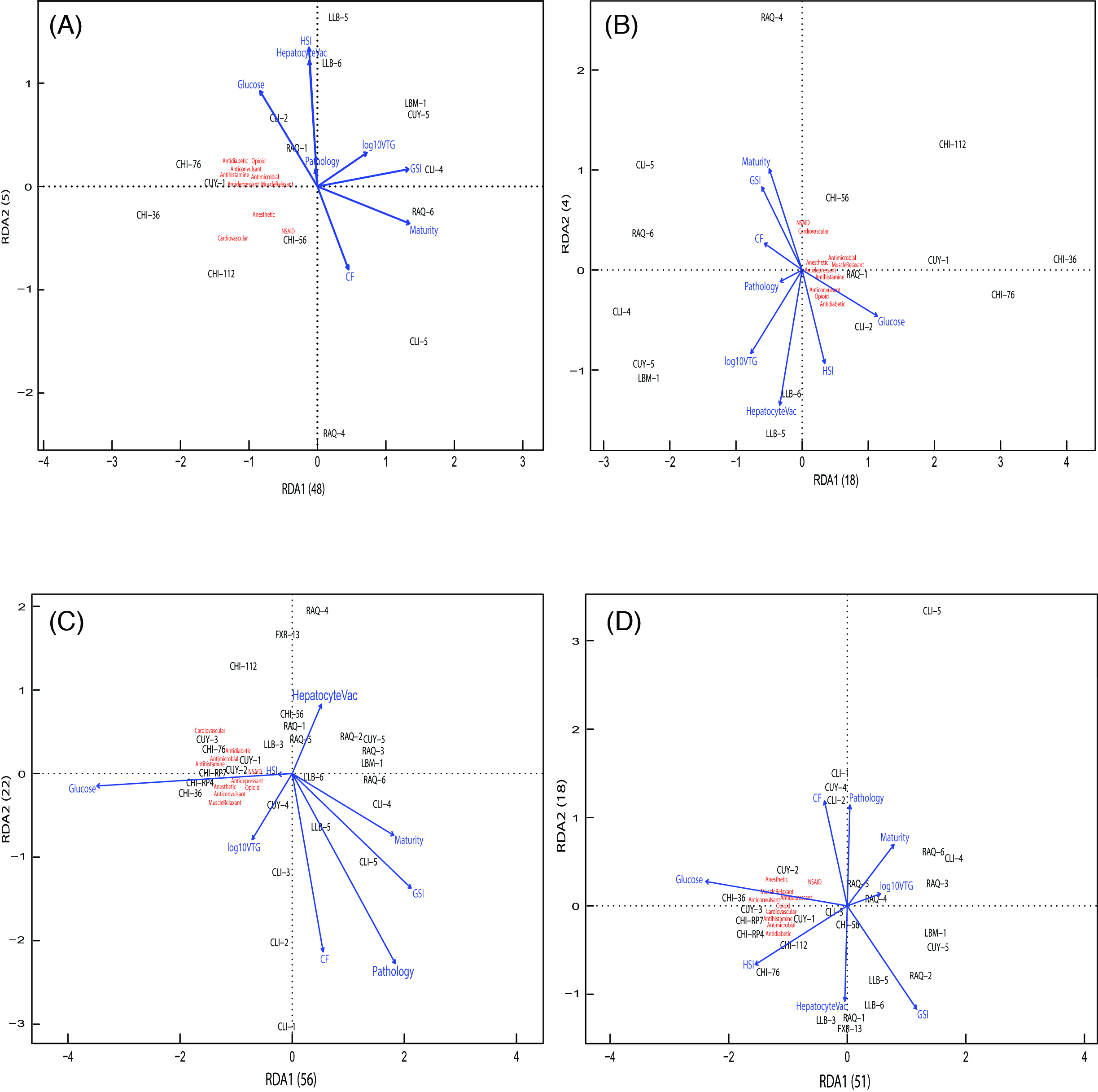

Supplement: S12 Fig — (A) Resident females, (B) Resident males, (C) Caged females, and (D) Caged males. Number in parentheses on axes indicate the percent variability that is explained by that axis. Sample site information can be found in Table 1. Sample subclass information can be found in S2 Table. Biological response information can be found in Table 4. (TIF) [file pone.0184725.s012.tif]
