# Supplementary material for: Contaminants of emerging concern in tributaries to the Laurentian Great Lakes: II. Biological consequences of exposure
Source: PLoS One. 2017 Sep 27;12(9):e0184725. doi: 10.1371/journal.pone.0184725 (PMC5617166; doi:10.1371/journal.pone.0184725)
Supplement: S1 File — R code developed in the Vegan software package for R. (DOCX) [file pone.0184725.s018.docx]

**Supplemental Code File (Microsoft Word file): R code developed in the Vegan software package for R.**

library(vegan)

setwd(“pathname”) # Here, pathname is the directory, for example C:/analysis.

data = read.csv("heiko_logCECsByClass_CagedFemales.csv", header=T, stringsAsFactors=F)

rownames(data)<-data$CagePos

data.all <- data[,c(4:43)]

data.all <- as.matrix(data.all) # per Jared: convert data frame to a matrix, so labels print

x <- data.all[,9:40]

y <- data.all[,1:8]

rda.all <- rda(x,y)

pdf("all_logCECsByClass_CagedFemales.pdf", width=10, height=10)

plot(rda.all, main="all_logCECsByClass_CagedFemales")

dev.off()

summary(rda.all)

forward.sel (y, x) # forward model selection

# ordistep model selection

# For ordistep and ordiR2step, R seems to require matrices be converted back to data frames.

x.df<-data.frame(x)

y.df<-data.frame(y)

mod0 <- rda(y.df ~ 1, x.df) ## Model with intercept only

mod1 <- rda(y.df ~ . ,x.df) # Model with all explanatory variables
